# Outcome prediction comparison of ischaemic areas’ radiomics in acute anterior circulation non-lacunar infarction

**DOI:** 10.1093/braincomms/fcae393

**Published:** 2024-11-15

**Authors:** Xiang Zhou, Jinxi Meng, Kangwei Zhang, Hui Zheng, Qian Xi, Yifeng Peng, Xiaowen Xu, Jianjun Gu, Qing Xia, Lai Wei, Peijun Wang

**Affiliations:** Department of Radiology, Tongji Hospital, School of Medicine, Tongji University, Shanghai 200065, China; Department of Radiology, Tongji Hospital, School of Medicine, Tongji University, Shanghai 200065, China; Department of Radiology, Tongji Hospital, School of Medicine, Tongji University, Shanghai 200065, China; Department of Radiology, Xinhua Hospital, School of Medicine, Shanghai Jiao Tong University, Shanghai 200092, China; Department of Radiology, Shanghai East Hospital, School of Medicine, Tongji University, Shanghai 200120, China; Department of Radiology, Putuo Hospital, Shanghai University of Traditional Chinese Medicine, Shanghai 200062, China; Department of Radiology, Tongji Hospital, School of Medicine, Tongji University, Shanghai 200065, China; Department of Radiology, Shanghai General Hospital, School of Medicine, Shanghai Jiao Tong University, Shanghai 200080, China; SenseTime Research, Shanghai 200232, China; Department of Radiology, Tongji Hospital, School of Medicine, Tongji University, Shanghai 200065, China; Department of Radiology, Tongji Hospital, School of Medicine, Tongji University, Shanghai 200065, China; Institute of Medical Imaging Artificial Intelligence, Tongji University School of Medicine, Shanghai 200065, China

**Keywords:** acute ischaemic stroke, magnetic resonance imaging, radiomics, model construction, prognosis prediction

## Abstract

The outcome prediction of acute anterior circulation non-lacunar infarction (AACNLI) is important for the precise clinical treatment of this disease. However, the accuracy of prognosis prediction is still limited. This study aims to develop and compare machine learning models based on MRI radiomics of multiple ischaemic-related areas for prognostic prediction in AACNLI. This retrospective multicentre study consecutively included 372 AACNLI patients receiving MRI examinations and conventional therapy between October 2020 and February 2023. These were grouped into training set, internal test set and external test set. MRI radiomics features were extracted from the mask diffusion-weighted imaging, mask apparent diffusion coefficient (ADC) and mask ADC620 by AACNLI segmentations. Grid search parameter tuning was performed on 12 feature selection and 9 machine learning algorithms, and algorithm combinations with the smallest rank-sum of area under the curve (AUC) was selected for model construction. The performances of all models were evaluated in the internal and external test sets. The AUC of radiomics model was larger than that of non-radiomics model with the same machine learning algorithm in the three mask types. The radiomics model using least absolute shrinkage and selection operator—random forest algorithm combination gained the smallest AUC rank-sum among all the algorithm combinations. The AUC of the model with ADC620 was 0.98 in the internal test set and 0.91 in the external test set, and the weighted average AUC in the three sets was 0.96, the largest among three mask types. The Shapley additive explanations values of the maximum of National Institute of Health Stroke Scale score within 7 days from onset (7-d NIHSS_max_), stroke-associated pneumonia and admission Glasgow coma scale score ranked top three among the features in AACNLI outcome prediction. In conclusion, the random forest model with mask ADC620 can accurately predict the AACNLI outcome and reveal the risk factors leading to the poor prognosis.

## Teaser

Zhou *et al.* report that the random forest model based on clinical, radiological and MRI radiomics features of multiple ischaemic masks can accurately predict the outcome of acute anterior circulation non-lacunar infarction. Severe neurological impairment within 7 days of onset and stroke-associated pneumonia indicates poor prognosis of acute ischaemic stroke.

## Graphical Abstract

**Graphical Abstract:**
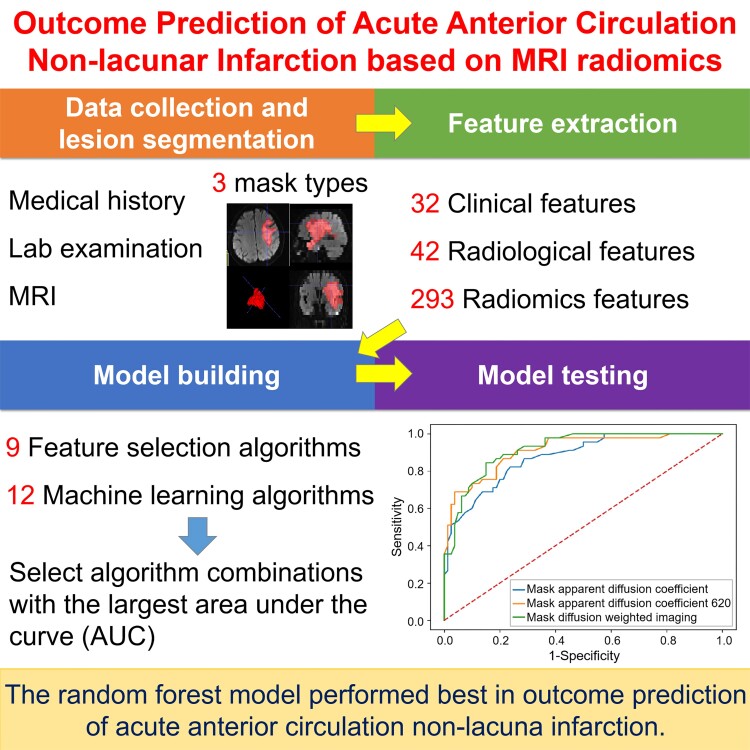

## Introduction

Acute ischaemic stroke (AIS) leads to hypoxic necrosis of brain tissue and neurological dysfunction worldwide.^1^ The incidence rate of non-lacunar infarction is 41.17% of all ischaemic stroke,^2^ and the prognosis of non-lacunar infarction is worse than that of lacunar infarction. Moreover, anterior circulation stroke account for 80% of all ischaemic strokes.^3^ Therefore, this study focused on acute anterior circulation non-lacunar infarction (AACNLI) to identify key factors leading to poor outcome and to improve the prognosis.

MRI is the most sensitive imaging methods for detecting AIS, providing important information about lesions, responsible arteries and collateral circulation. Diffusion-weighted imaging (DWI) can reflect the movement of water molecules, sensitive to early cerebral infarction. Most studies regarded DWI high signal as the infarct region, but DWI high-signal reversal in AIS has been found in minor stroke.^4^ At present, the threshold of apparent diffusion coefficient (ADC) < 620 × 10^−6^ mm²/s is adopted as the standard for ischaemic core.^5^ However, there still exists the region of ADC < 620 × 10^−6^ mm²/s with DWI normal appearance adjacent to ischaemic core. The correlation between features of ischaemic core with its adjacent region and prognosis deserves further research. Therefore, this study explored three ischaemic lesion segmentation methods (DWI high-signal method, ischaemic core method and ischaemic core with its adjacent region method) to select the optimal method for AACNLI outcome prediction.

Radiomics is an emerging technology used to extract much information from image data for predicting outcomes.^6^ At present, there are some studies extracting MRI radiomics features for AIS outcome prediction.^7-9^ Most studies used least absolute shrinkage and selection operator (LASSO) as the radiomics feature selection (FS) method, without comparing multiple FS algorithms. Machine learning (ML) algorithm is suitable for analysing rules from data and classifying unknown data.^7,10^ At present, most studies used only a few ML algorithms for model construction, such as logistic regression, support vector machine and random forest (RF), without comparing other ML algorithms. Therefore, this study used more radiomics FS algorithms and ML algorithms to select the optimal algorithm combination for model construction, which can improve the accuracy of AACNLI outcome prediction.

The study aims to explore the combination of multiple FS and ML algorithms to build models to predict the clinical outcome of AACNLI by mining the radiomics, radiological and clinical features of different ischaemic regions, so as to find the high-risk factors leading to poor prognosis and provide an important basis for clinical precision treatment of AACNLI.

## Methods

### Institutional review board statement

The study was conducted according to the guidelines of the Declaration of Helsinki and approved by the Institutional Review Board: Ethics Committee of Tongji Hospital (approval number: K-2020-021; date of approval: 20 November 2020).

### Informed consent statement

The need for written informed consent was waived by Ethics Committee of Tongji Hospital.

### Study population

This multicentre retrospective study consecutively collected clinical, imaging and follow-up data of AACNLI patients admitted to Tongji Hospital, Xinhua Hospital, Shanghai General Hospital, Dongfang Hospital and Putuo Hospital from October 2020 to February 2023.

Inclusion criteria included the following: (i) MRI and clinical diagnosis of AIS in the anterior circulation; (ii) age ≥ 18 years old, no previous stroke or previous stroke without neurological impairment; (iii) MRI and computed tomography angiography completed within 72 h after onset; (iv) MRI showed AACNLI (maximum diameter ≥ 15 mm); (v) AIS patients received hospitalized antiplatelet treatment within 24 h after onset.^11^

Exclusion criteria included the following: (i) the interval between the last stroke and the current stroke < 6 months; (ii) recent myocardial infarction (<3 weeks), malignant tumours *in vivo*, brain tumours, brain trauma, moyamoya disease or history of brain surgery; (iii) clinical contraindications to MRI; (iv) decreased image quality due to the motion or flow artefacts; (v) haemorrhagic cerebral infarction or anterior with posterior circulation infarction; (vi) patients received thrombolysis, thrombectomy or surgical treatments; (vii) patients with incomplete clinical and follow-up information.

We enrolled 247 AACNLI patients admitted to Tongji Hospital, Xinhua Hospital and Shanghai General Hospital from October 2020 to September 2022 and divided these patients’ data into training set (*n* = 172) and internal test set (*n* = 75) by stratifying random sampling in a 7:3 ratio. We also enrolled 125 AACNLI patients admitted to Dongfang Hospital and Putuo Hospital from October 2022 to February 2023 as external test set (Supplementary Fig. 1). The data supporting the findings of this study are available from the corresponding author upon reasonable request.

### Data collection

The clinical data were collected as follows: (i) gender and age; (ii) history of smoking, alcohol consumption, diabetes, myocardial infarction, coronary atherosclerosis, atrial fibrillation, hypertension, stroke, heart failure, hyperlipidaemia and hyperhomocysteinaemia; (3) secondary cognitive impairment, epilepsy and stroke-associated pneumonia (SAP); (4) admission systolic blood pressure (SBP) and diastolic blood pressure; (5) admission National Institute of Health Stroke Scale (NIHSS) and Glasgow Coma Scale (GCS) scores; (6) the maximum of NIHSS score within 7 days from onset (7-d NIHSS_max_); (7) admission laboratory examinations; (8) the Trial of Org10172 in Acute Stroke Treatment type and Oxfordshire Community Stroke Project (OCSP) type.

This study used 3.0 T MRI imaging equipment (Philips Medical Systems INGENIA, SIEMENS Verio, United Imaging uMR 770) and 8-channel head coil to collect brain signals. The scanning range was from the bottom of the posterior cranial fossa to the top of the skull (details in Supplementary Table 1). This study also used CT equipment (Philips Brilliance iCT, Toshiba Aquilion ONE, United Imaging uCT780) to collect head and neck computed tomography angiography images. Iodine contrast agent 50–60 ml and normal saline 30 ml were injected successively at the injection rate of 5 ml/s. The scanning range was from the aortic arch to the skull top. The scanning parameters were as follows: 120 kV, 198–282 mAs, slice thickness = 0.75 mm, slice spacing = 0.7 mm, matrix = 512 × 512.

The follow-up data were collected by querying the hospital outpatient medical record system or conducting telephone follow-up. Three-month modified Rankin Scale (mRS) score was performed to measure the neurological recovery after stroke, with 0–2 indicating good prognosis and 3–6 indicating poor prognosis.^12^

### Image analysis and AIS lesion segmentation

The radiological features, including admission Fazekas score,^13^ DWI-Alberta Stroke Program Early CT Score,^14^ clot-based score,^15^ DWI fluid-attenuated inversion recovery (FLAIR) mismatch,^16^ FLAIR vascular hyperintensity score,^17,18^ DWI-FLAIR vascular hyperintensity mismatch^19^ and haemorrhage transformation were analysed by a radiologist with 10-year experience in neuroradiology (details in Supplementary Appendix 1).

The segmentation task was completed with ITK-SNAP software (version 3.8.0, http://www.itksnap.org) by two neuroradiologists (X.Z. and L.W. with 10 and 12 years of experience, respectively) independently. Another radiologist (Y.P.) with 26 years of experience in neuroradiology examined the segmentation results. As for the mask DWI, the AIS lesion was segmented along a high signal on DWI slice by slice. As for the mask ADC, the region with ADC threshold of (0–620) × 10^−6^ mm^2^/s in the mask DWI was segmented on the ADC map. As for the mask ADC620, mask ADC and its adjacent region with ADC threshold of (0–620) × 10^−6^ mm^2^/s was segmented on the ADC map, avoiding the normal basal ganglia nuclei and subcortical regions (Supplementary Fig. 2). The radiological features of masks were measured and calculated (details in Supplementary Appendix 2).

### Radiomics feature extraction, selection and modelling

The N4 bias field correction and normalization were applied to process the MRI pixels’ greyscale, and the matrix of images was resampled to 256 × 256. DWI was selected as a reference to perform spatial position registration on T2-FLAIR fat-suppression images. ADC images were calculated from DWI.

Pyradiomics software package was applied to extract radiomics features from the masks.^20^ One hundred and seven radiomics features were extracted from DWI, ADC maps and T2-FLAIR fat-suppression images, respectively, which fundamentally met the Image Biomarker Standardization Initiative standard. After the removal of 28 repetitive and collinear radiomics features, the remaining 293 radiomics features were screened by 12 FS algorithms.

Nine ML algorithms were used to construct the model with clinical + radiological features and clinical + radiological + radiomics features, respectively. Grid search and 5-fold cross-validation by using training data were performed on 108 FS-ML algorithm combinations for parameter tuning.^21^ The AUC of algorithm combination of three mask types were calculated and ranked, respectively. However, the algorithm combination with the smallest rank-sum was selected. For the model with clinical + radiological features, the ML algorithm was the same as which was used in the model with clinical + radiological + radiomics features. The principal codes about radiomics feature extraction, FS and modelling are shown in Supplementary Appendix 3.

### Model test and evaluation

Receiver operating characteristic curve was drawn, and the sensitivity, specificity, accuracy, AUC, precision and F1 score were calculated to evaluate the performance of the model. The calibration curve was drawn to evaluate the consistency between the predicted and actual observed results. The decision analysis curve was drawn to evaluate the clinical practicability of the model. A Shapley additive explanations (SHAP) diagram was plotted for model interpretation.^21^

### Statistical analysis

SPSS 20.0 and MedCalc 20.019 statistical software was applied to analyse the data. Student’s *t*-test, Mann–Whitney *U* test and Pearson *χ*^2^ test were applied to compare the variables. Multicollinearity diagnosis was performed to eliminate highly correlated multiple independent variables. The diagnostic significance between the models was evaluated using the DeLong test. The difference was statistically significant at *P* ≤ 0.05. Heatmaps were plotted to show the AUC results of FS–ML algorithm combinations of three mask types. AUC > 0.75 indicates better prediction performance.^22^ The Spearman correlation was performed to analyse the relationship between radiomics features and mRS score. The task of training and test of the model were performed by Python 3.7.

## Results

### Patient characteristics

Among the 247 AACNLI patients in the training set and internal test set, 132 patients had good prognosis and 115 patients had poor prognosis. There existed no significant differences between the training set and internal test set in the clinical and radiological features (Supplementary Table 2). For the clinical features, there existed significant differences between the two groups in gender, age, history of smoking, coronary atherosclerosis, atrial fibrillation, heart failure, hyperlipidaemia, SAP, admission SBP, Trial of Org10172 in Acute Stroke Treatment type, OCSP type, admission NIHSS score, admission GCS score, 7-d NIHSS_max_, CRP, PT, D-dimer, serum troponin I, blood glucose and plasma BNP (Table 1). For the radiological features, there existed statistically significant differences between good and poor prognosis groups in clot-based score, DWI-Alberta Stroke Program Early CT Score, mask DWI-related features (V1, V1_max_, P1, ADC1_SD_, ADC1_CV_, Grad1_CV_, rADC1), mask ADC620-related features (V2, V2_max_, P2, ADC2_mean_, ADC2_SD_, ADC2_CV_, Grad2_SD_, Grad2_CV_, rADC2) and mask ADC-related features (V3, V3_max_, P3, N3, ADC3_mean_, ADC3_SD_, ADC3_CV_, Grad3_SD_, Grad3_CV_, rADC3; Table 2).

**Table 1:** Clinical features of AACNLI patients with good and poor outcome (training set + internal test set)

Features	Good Outcome (*n* = 132)	Poor Outcome (*n* = 115)	*P-*value
Demography			
Female	46 (34.8%)	58 (50.4%)	0.013
Age	69 (62, 77)	76 (65, 85)	0.002
History			
Smoking	73 (55.3%)	40 (34.8%)	0.001
Alcohol consumption	41 (31.1%)	25 (21.7%)	0.099
Diabetes	45 (34.1%)	35 (30.4%)	0.540
Myocardial infarction	1 (0.8%)	5 (4.3%)	0.068
Coronary atherosclerosis	17 (12.9%)	34 (29.6%)	0.001
Atrial fibrillation	21 (15.9%)	41 (35.7%)	<0.001
Hypertension	90 (68.2%)	84 (73.0%)	0.404
Stroke	29 (22.0%)	37 (32.2%)	0.071
Heart failure	13 (9.8%)	30 (26.1%)	0.001
Hyperlipidaemia	21 (15.9%)	7 (6.1%)	0.015
Hyperhomocysteinaemia	1 (0.8%)	4 (3.5%)	0.288
Secondary disease			
Cognitive impairment	2 (1.5%)	5 (4.3%)	0.340
Epilepsy	3 (2.3%)	3 (2.6%)	>0.999
SAP	8 (6.1%)	62 (53.9%)	<0.001
Blood pressure on admission			
SBP (mmHg)	146.66 (20.93)	152.49 (21.90)	0.034
DBP (mmHg)	81.5 (77, 90)	80 (78, 90)	0.984
TOAST type			<0.001
Large-artery atherosclerosis	95 (72.0%)	80 (69.6%)	
Cardioembolism	17 (12.9%)	31 (27.0%)	
Small-artery occlusion	17 (12.9%)	2 (1.7%)	
Other determined aetiology	3 (2.3%)	2 (1.7%)	
OCSP type			<0.001
TACI	3 (2.3%)	41 (35.7%)	
PACI	127 (96.2%)	72 (62.6%)	
LACI	2 (1.5%)	2 (1.7%)	
Neurological scale score			
Admission NIHSS	4 (2, 6)	13 (8, 19)	<0.001
Admission GCS	15 (15, 15)	13 (11, 15)	<0.001
7d NIHSS_max_	4 (2, 6)	15 (12, 19)	<0.001
Laboratory test			
CRP (mg/l)	3.05 (1.27, 6.80)	8.33 (2.50, 22.41)	<0.001
PLT (×10^9^/l)	200 (166, 243)	199 (156, 240)	0.680
PT (s)	11.1 (10.6, 11.7)	11.3 (10.8, 11.9)	0.021
Fibrinogen (g/l)	2.85 (2.44, 3.26)	2.95 (2.55, 3.70)	0.070
D-dimer (mg/l)	0.41 (0.27, 0.79)	0.86 (0.41, 1.79)	<0.001
Serum troponin I (ng/ml)	0.010 (0.008, 0.012)	0.013 (0.010, 0.030)	<0.001
Blood sugar (mmol/l)	6.28 (5.24, 7.96)	6.73 (5.88, 9.24)	0.009
Triglyceride (mmol/l)	1.22 (0.95, 1.65)	1.22 (0.94, 1.64)	0.094
Plasma BNP (pg/ml)	73.7 (38.25, 156.4)	133.0 (59.9, 356.7)	<0.001
Follow-up			
3-month mRS	1 (1, 2)	4 (3, 4)	<0.001

All categorical variables are expressed as *n* (%) and continuous variables as median (IQR) or mean (SD). BNP, brain natriuretic peptide; CRP, C-reactive protein; DBP, diastolic blood pressure; GCS, Glasgow coma scale; IQR, interquartile range; LACI, lacunar infarction; mRS, modified Rankin Scale; NIHSS, National Institute of Health Stroke Scale; OCSP, Oxfordshire Community Stroke Project; PACI, partial anterior circulation infarction; PLT, platelet count; PT, prothrombin time; SAP, stroke-associated pneumonia; SBP, systolic blood pressure; SD, standard deviation; TACI, total anterior circulation infarction; TOAST, Trial of Org10172 in Acute Stroke Treatment.

**Table 2:** Radiological features of AACNLI patients with good and poor outcome (training set + internal test set)

Features	Good Outcome (*n* = 132)	Poor Outcome (*n* = 115)	*P*-value
Interval from onset to MRI (h)	35 (22, 50.5)	36 (21, 53)	0.975
CBS	10 (8, 10)	8 (6, 10)	<0.001
HT	13 (9.8%)	21 (18.3%)	0.056
DWI-ASPECTS	7 (6, 8)	5 (3, 7)	<0.001
Contralateral brain ADC (×10^−6^ mm^2^/s)	777 (50)	784 (53)	0.254
DWI-FLAIR mismatch	4 (3.0%)	7 (6.1%)	0.245
DWI-FVH mismatch	92 (69.7%)	69 (60.0%)	0.111
FVH score	2 (1, 4)	3 (1, 4)	0.357
Fazekas score	2 (2, 3)	3 (2, 4)	0.092
Mask DWI			
V1 (ml)	10.29 (4.93, 23.57)	46.99 (15.05, 149.04)	<0.001
V1max (ml)	8.84 (3.80, 19.06)	41.16 (13.08, 149.04)	<0.001
P1	0.87 (0.68, 0.99)	0.99 (0.93, 1.00)	<0.001
N1	6 (2, 11)	4 (2, 10)	0.095
ADC1mean (×10^−6^ mm^2^/s)	599 (537, 666)	565 (507, 640)	0.063
ADC1SD (×10^−6^ mm^2^/s)	177 (145, 221)	193 (162, 252)	0.013
ADC1CV	0.31 (0.25, 0.35)	0.34 (0.29, 0.41)	<0.001
Grad1mean	8576 (6665, 11047)	8665 (7161, 10839)	0.659
Grad1SD	8112 (5486, 11109)	9144 (7100, 11424)	0.070
Grad1CV	0.92 (0.79, 1.06)	1.04 (0.89, 1.16)	<0.001
rADC1	0.75 (0.68, 0.87)	0.72 (0.64, 0.83)	0.018
Mask ADC620			
V2 (ml)	5.81 (3.31, 14.58)	34.68 (10.16, 115.82)	<0.001
V2max (ml)	4.91 (2.25, 13.42)	34.60 (7.08, 114.78)	<0.001
P2	0.90 (0.64, 0.98)	0.99 (0.88, 1.00)	<0.001
N2	7 (3, 12)	9 (3, 16)	0.108
ADC2mean (×10^−6^ mm^2^/s)	496 (40)	477 (40)	<0.001
ADC2SD (×10^−6^ mm^2^/s)	75 (61, 95)	89 (74, 100)	0.001
ADC2CV	0.15 (0.12, 0.20)	0.19 (0.15, 0.22)	0.001
Grad2mean	7336 (6017, 9726)	7856 (6648, 9301)	0.237
Grad2SD	6741 (4574, 9561)	8153 (6440, 10203)	0.003
Grad2CV	0.88 (0.74, 1.04)	1.06 (0.85, 1.15)	<0.001
rADC2	0.64 (0.06)	0.61 (0.06)	<0.001
Mask ADC			
V3 (ml)	5.32 (2.87, 13.07)	31.76 (8.22, 110.61)	<0.001
V3max (ml)	4.11 (1.79, 11.10)	30.61 (5.94, 109.46)	<0.001
P3	0.88 (0.63, 0.99)	0.99 (0.86, 1.00)	<0.001
N3	8 (4, 14)	10 (4, 21)	0.049
ADC3mean (×10^−6^ mm^2^/s)	499 (455, 526)	469 (446, 508)	0.003
ADC3SD (×10^−6^ mm^2^/s)	75 (62, 94)	90 (72, 99)	0.001
ADC3CV	0.15 (0.12, 0.21)	0.19 (0.15, 0.22)	0.001
Grad3mean	7336 (5986, 9529)	7707 (6481, 9069)	0.448
Grad3SD	6804 (4644, 9595)	8117 (6373, 10033)	0.007
Grad3CV	0.89 (0.74, 1.05)	1.07 (0.89, 1.19)	<0.001
rADC3	0.63 (0.07)	0.60 (0.06)	<0.001

All categorical variables are expressed as *n* (%) and continuous variables as median (IQR) or mean (SD).

ADC, apparent diffusion coefficient; ASPECTS, Alberta Stroke Program Early CT Score; CBS, clot burden score; CV, coefficient of variation; DWI, diffusion-weighted imaging; FLAIR, fluid-attenuated inversion recovery; FVH, FLAIR vascular hyperintensity; Grad, gradient; HT, haemorrhage transformation; IQR, interquartile range; *N*, number of the lesions; *P*, proportion (Vmax/V); SD, standard deviation; V, total volume of the lesions; Vmax, volume of the largest lesion.

Among the 125 AACNLI patients in the external test set, 80 patients had good prognosis and 45 patients had poor prognosis. The detailed clinical and radiological features are shown in Supplementary Tables 3 and 4.

### Algorithm selection for AACNLI prognostic prediction model

**Figure 1:**
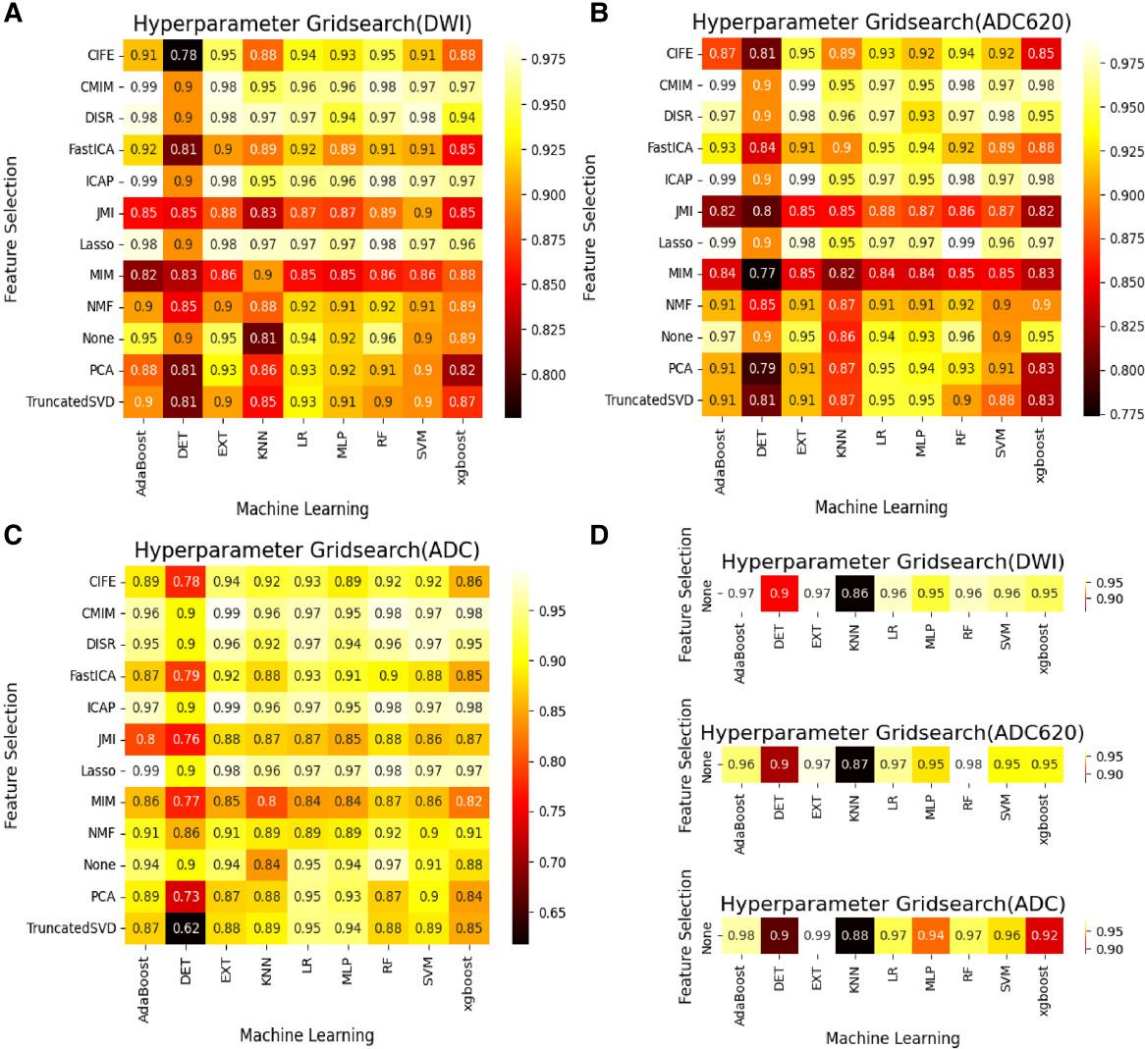
**Hyperparameter grid search of the FS and ML algorithm combinations by using training data with 5-fold cross-validation.** (**A–C**) The AUC results of FS and ML algorithm combinations based on clinical + radiological + radiomics variables with (**A**) mask DWI, (**B**) mask ADC620 and (**C**) mask ADC. (**D**) The AUC results of ML algorithm based on clinical + radiological variables with the above three types of masks. Adaboost, adaptive boosting; CIFE, common and individual feature extraction; CMIM, conditional mutual information maximization; DET, deep extremely randomized trees; DISR, dental image segmentation and retrieval; EXT, extremely randomized trees; Fast ICA, fast independent component analysis; ICAP, interaction capping; JMI, joint mutual information; KNN, K-nearest neighbour; MIM, mutual information maximization; MLP, multi-layer perceptron; NMF, non-negative matrix factorization; None, all features without any selection; PCA, principal component analysis; Truncated SVD, truncated singular value decomposition; Xgboost, extreme gradient boosting.

Based on clinical + radiological + radiomics features of training set, 12 FS algorithms and 9 ML algorithms were used to construct the model (Supplementary Tables 5 and 6). After 5-fold cross-validation by using training data, the AUC grid search results of algorithm combination are shown in Fig. 1A–C. The AUC results of the LASSO-RF algorithm combination were 0.98, 0.99 and 0.98 for mask DWI (rank third), mask ADC620 (rank first) and mask ADC (rank fourth), respectively. The rank-sum of LASSO-RF algorithm combination was the smallest (rank-sum of 8) among all the algorithm combinations, indicating the best performance.

Based on the clinical + radiological features of training set, 9 ML classification algorithms were used to construct the model, and the AUC results are shown in Fig. 1D. The AUC of radiomics model with the optimal FS-ML algorithm combination was larger than that of non-radiomics model with the same ML algorithm in the three mask types (Supplementary Table 7). Therefore, this study used clinical + radiological + radiomics features to construct the model.

### Feature selection and model construction

Based on the sparsity hypothesis, LASSO regression eliminates continuous or discrete variables with a regression coefficient of 0, controlling the degree of contraction constraint by finding the coefficient *α* of the norm. The LASSO errors and paths of the three mask types are demonstrated in Supplementary Fig. 3.

For the mask DWI, 27 selected radiomics features (Supplementary Table 8), 12 selected clinical features (age, smoking history, history of hyperlipidaemia, SAP, SBP at the first diagnosis, OCSP type, admission GCS score, 7-d NIHSS_max_, prothrombin time, D-dimer, serum troponin I, blood glucose) and 2 selected radiological features (ADC1_CV_, Grad1_CV_) were included in the model.

For the mask ADC620, 28 selected radiomics features (Supplementary Table 9), 12 selected clinical features (age, smoking history, history of hyperlipidaemia, SAP, SBP at the first diagnosis, Trial of Org10172 in Acute Stroke Treatment type, OCSP type, admission GCS score, 7-d NIHSS_max_, D-dimer, serum troponin I, blood glucose) and two selected radiological features (Grad2_CV_, rADC2) were included in the model.

For the mask ADC, 27 selected radiomics features (Supplementary Table 10), 12 selected clinical features (age, smoking history, history of hyperlipidaemia, SAP, SBP at the first diagnosis, OCSP type, admission GCS score, 7-d NIHSS_max_, CRP, D-dimer, serum troponin I, blood glucose) and 2 selected radiological features (Grad3_CV_, clot-based score) were included in the model.

### Model interpretation

**Figure 2:**
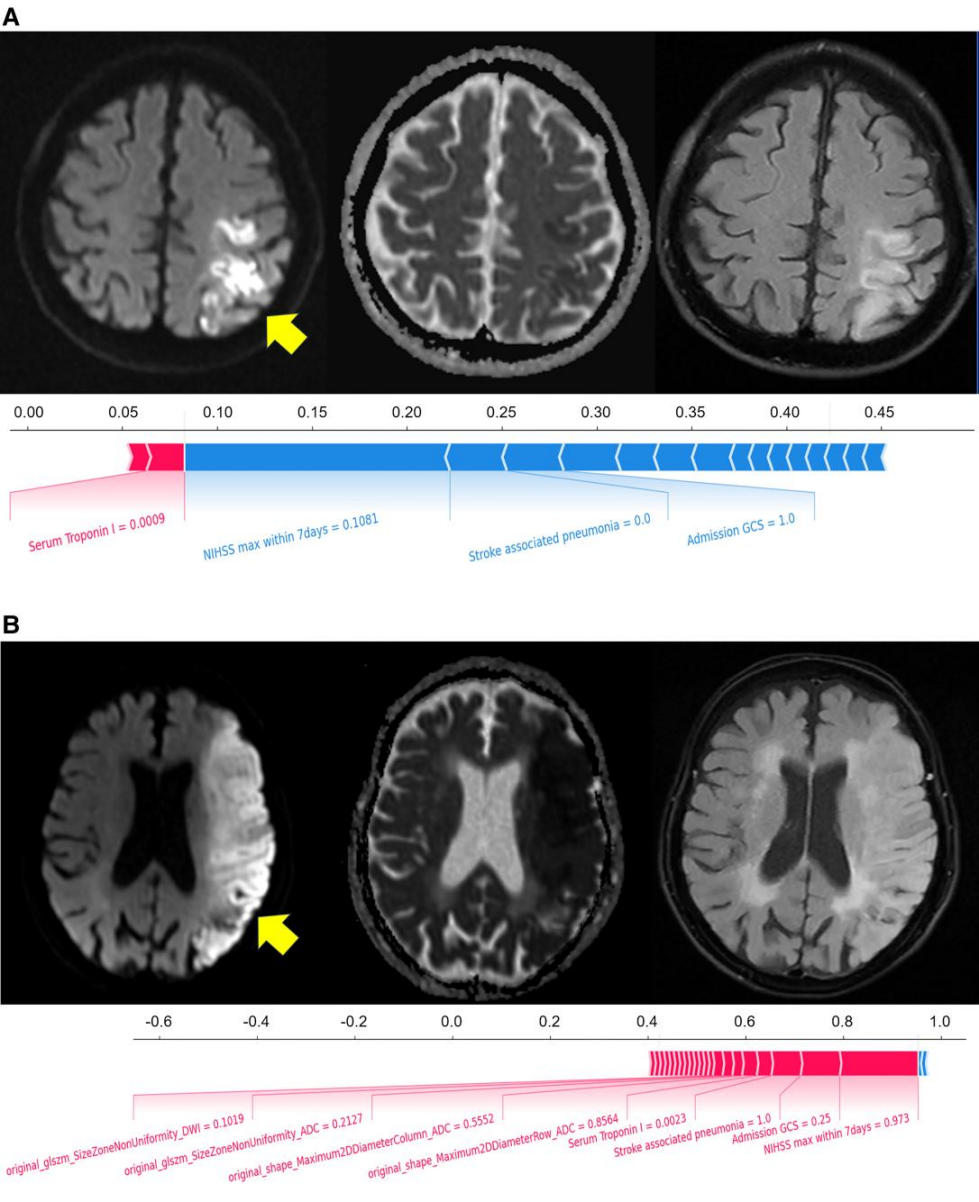
**SHAP force plots of the AACNLI patients with good and poor outcome.** (**A**) A 71-year-old male patient with 7-d NIHSS_max_ of 5- and 3-month mRS score of 2 showed a patchy AIS in the left parietal lobe on DWI (arrow), with ADC_mean_ of 523 × 10^−6^ mm^2^/s and high signal on FLAIR fat-suppression image. (**B**) A 68-year-old female patient with 7-d NIHSS_max_ of 17- and 3-month mRS score of 5 showed large AIS in the left temporal and occipital lobe on DWI (arrow), with ADC_mean_ of 478 × 10^−6^ mm^2^/s and slightly high signal on FLAIR fat-suppression image. The SHAP force plots of both patients show that 7-d NIHSS_max_, admission GCS score and SAP are weighted higher than other features influencing the prognosis. glszm, grey-level size zone matrix.

**Figure 3:**
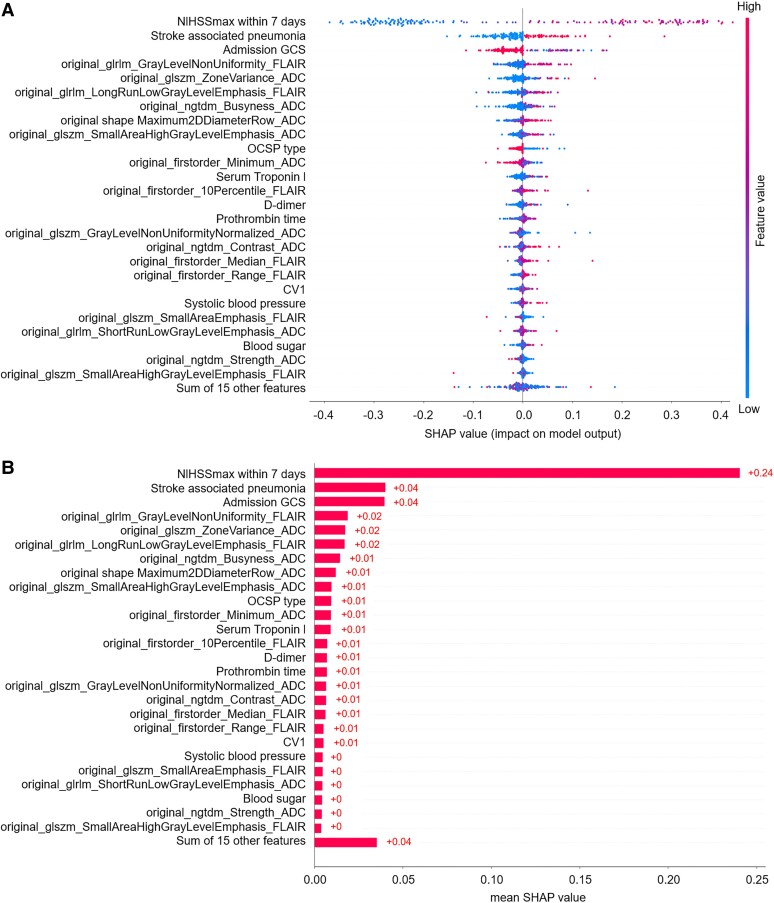
**SHAP beeswarm charts and global bar plots of the prognostic prediction model based on mask DWI.** (**A**) SHAP beeswarm chart of mask DWI shows SHAP values of each feature as scatter. (**B**) SHAP global bar plots of mask DWI shows the averaged SHAP values of each feature, which indicate the importance of each feature in the model. CRP, C-reactive protein; CV, coefficient of variation; glcm, grey-level cooccurrence matrix; gldm, grey-level difference matrix; glrlm, grey-level run-length matrix; glszm, grey-level size zone matrix; ngtdm, neighbourhood grey-tone difference matrix.

**Figure 4:**
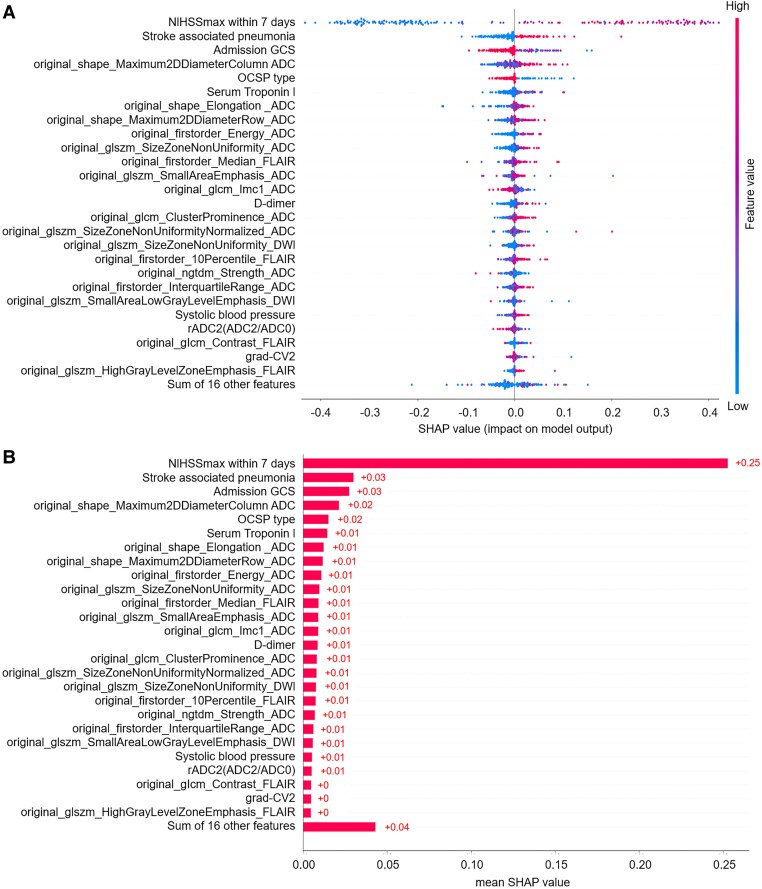
**SHAP beeswarm charts and global bar plots of the prognostic prediction model based on mask ADC620.** (**A**) SHAP beeswarm charts of mask ADC620 shows SHAP values of each feature as scatter. (**B**) SHAP global bar plots of mask ADC620 shows the averaged SHAP values of each feature. CRP, C-reactive protein; CV, coefficient of variation; glcm, grey-level cooccurrence matrix; gldm, grey-level difference matrix; glrlm, grey-level run-length matrix; glszm, grey-level size zone matrix; grad, gradient; ngtdm, neighbourhood grey-tone difference matrix; rADC, relative apparent diffusion coefficient.

**Figure 5:**
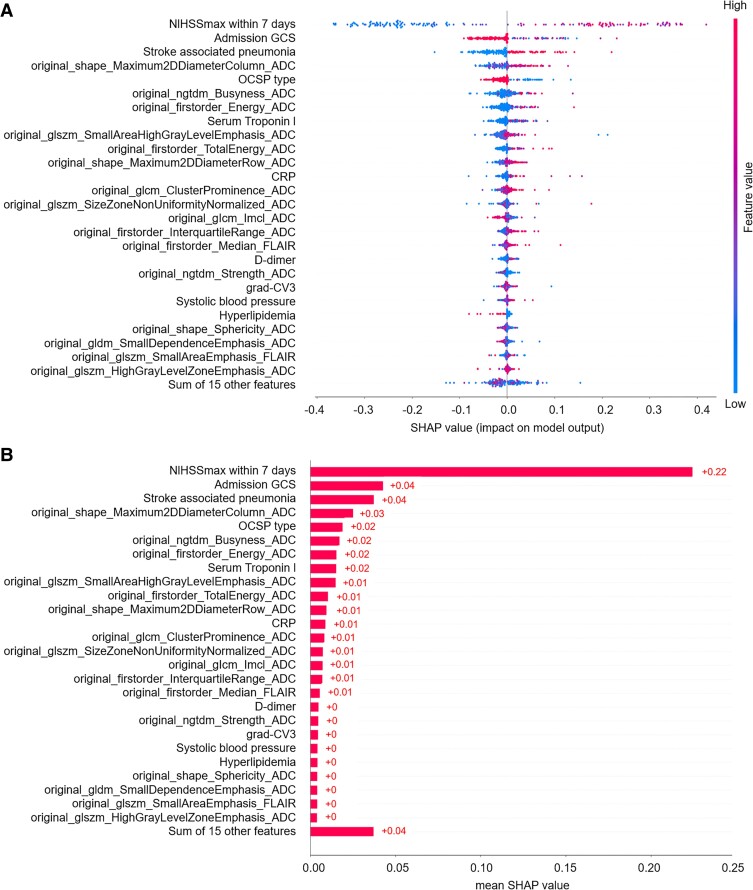
**SHAP beeswarm charts and global bar plots of the prognostic prediction model based on mask ADC.** (**A**) SHAP beeswarm charts of mask ADC shows SHAP values of each feature as scatter. (**B**) SHAP global bar plots of mask ADC shows the averaged SHAP values of each feature. CRP, C-reactive protein; CV, coefficient of variation; glcm, grey-level cooccurrence matrix; gldm, grey-level difference matrix; glrlm, grey-level run-length matrix; glszm, grey-level size zone matrix; grad, gradient; ngtdm, neighbourhood grey-tone difference matrix.

The SHAP diagram show the distribution of SHAP values for each feature over the total sample prediction. The detailed SHAP values of the most important features for good and poor outcome are illustrated in Fig. 2A and B. As for the RF model of mask DWI, the SHAP values of 7-d NIHSS_max_ (0.24), SAP (0.04) and admission GCS score (0.04) ranked top 3 among the features (Fig. 3A and B). As for the RF model of mask ADC620, the SHAP values of 7-d NIHSS_max_ (0.25), SAP (0.03) and admission GCS score (0.03) ranked top 3 among the features (Fig. 4A and B). As for the RF model of mask ADC, the SHAP values of 7-d NIHSS_max_ (0.22), SAP (0.04) and admission GCS score (0.04) ranked top 3 among the features (Fig. 5A and B).

### Model test and evaluation

**Figure 6:**
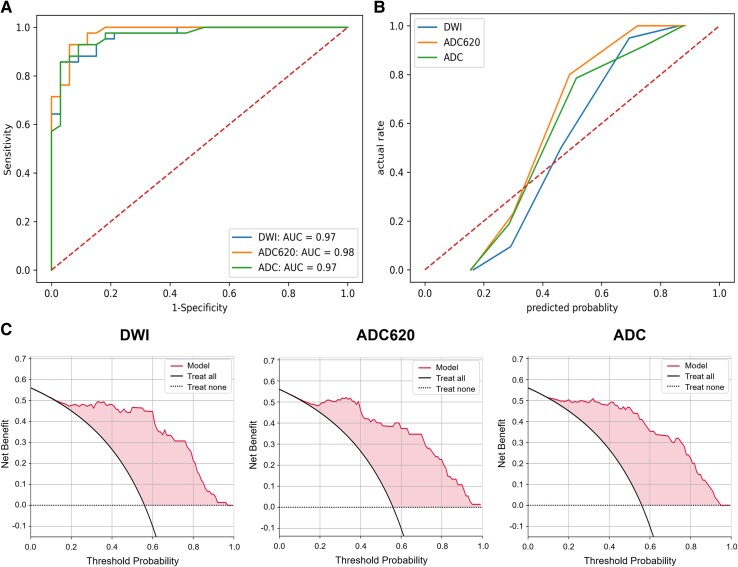
**Evaluation of the RF model in the internal test set.** (**A**) ROC curve, (**B**) calibration curve and (**C**) decision curves of RF models with three mask types in the internal test set. The task of model testing was performed by Python 3.7. Functions such as roc_curve, precision_recall_curve and decision_function in Python's ‘sklearn. metrics library’ were applied to calculate the required data points for the curves.

**Figure 7:**
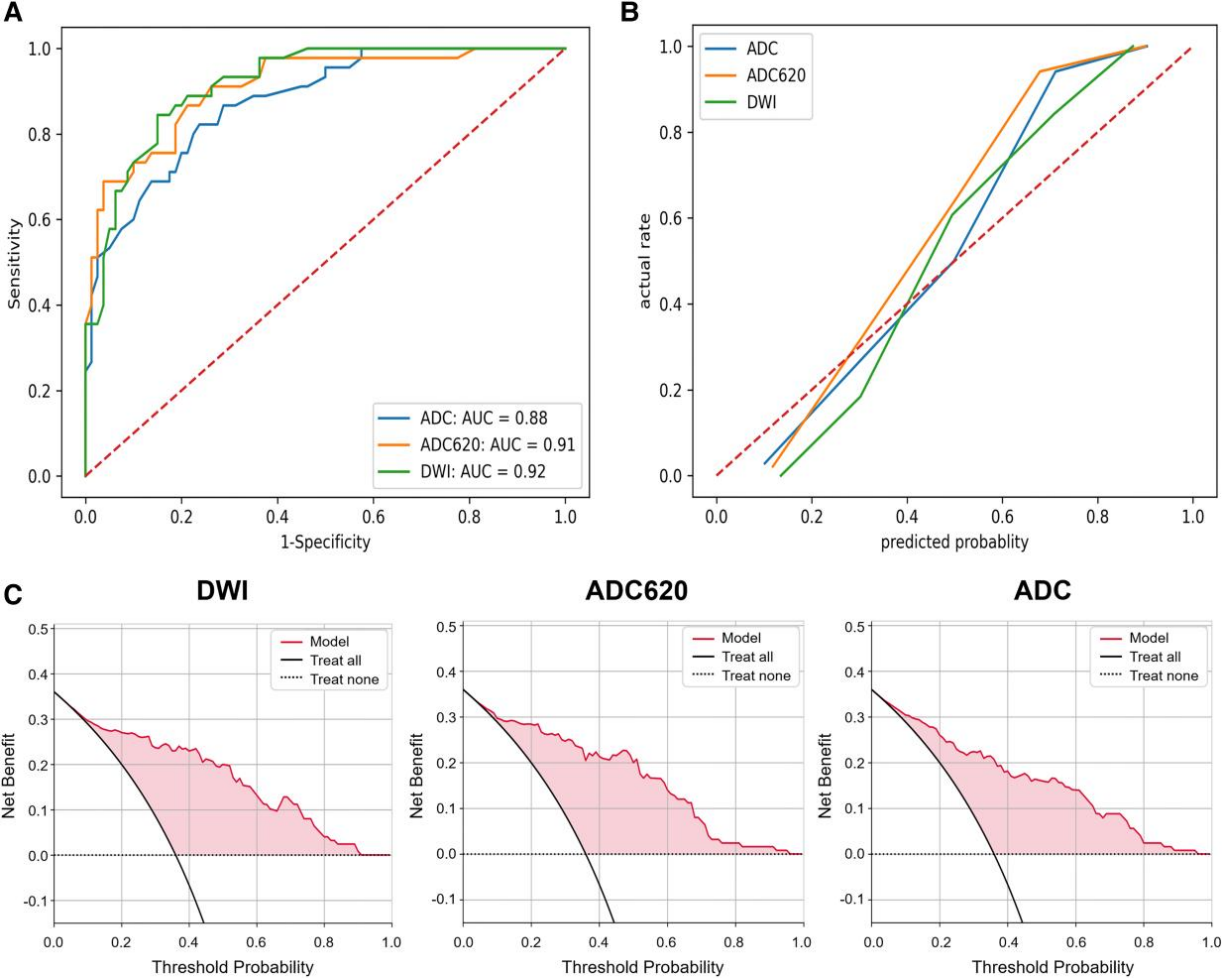
**Evaluation of the RF model in the external test set**. (**A**) ROC curve, (**B**) calibration curve and (**C**) decision curves of RF models with three mask types in the external test set. The task of model testing was performed by Python 3.7. Functions such as roc_curve, precision_recall_curve and decision_function in Python's ‘sklearn. metrics library’ were applied to calculate the required data points for the curves.

In the internal test set, the RF model’s AUC (0.98) of mask ADC620 was the largest among the three mask types (Fig. 6A). The accuracy (0.91) and F1 value (0.92) of the model with mask ADC were the highest. In the external test set, the RF model’s AUC (0.92) of mask DWI was the largest (Fig. 7A). The accuracy of model of mask DWI (0.84) and F1 value (0.79) were the highest. The calibration curves of internal test set (Fig. 6B) and external test set (Fig. 7B) showed good predictive accuracy between the actual probability and predicted probability. The decision curves of internal test set (Fig. 6C) and external test set (Fig. 7C) showed that intervention for high-risk patients with poor prognosis with a threshold probability of 0.2–0.9 will yield net benefit.

### AUC comparison of the three mask types

For the RF model of mask DWI, the AUCs of the training set (*n* = 172), internal test set (*n* = 75) and external test set (*n* = 125) were 0.98, 0.97 and 0.92, respectively. The weighted average AUC based on the number of cases in the three sets was 0.958. For the RF model of mask ADC620, the AUCs of the three sets were 0.99, 0.98 and 0.91, respectively, and the weighted average AUC was 0.961. For the RF model of mask ADC, the AUCs of the three sets were 0.98, 0.97 and 0.88, respectively, and the weighted average AUC was 0.944 (Table 3). Above all, the RF model of mask ADC620 had the largest weighted average AUC, indicating the best performance for prognostic prediction. In the model of mask ADC620, 11 of 28 radiomics features were moderately correlated with the 3-month mRS score with Spearman rank-order correlation coefficient ︱*r*︱= 0.4–0.6 (*P*<0.001; Supplementary Table 11).

**Table 3:** The performance of RF models with three masks in the training, internal test and external test set

Mask type	AUC (95%CI)	Sensitivity	Specificity	Accuracy	Precision	F1 value
Training set						
Mask DWI	0.98 (0.95, 1.00)	0.95	0.85	0.91	0.89	0.92
Mask ADC620	0.99 (0.96, 1.00)	0.95	0.88	0.92	0.91	0.93
Mask ADC	0.98 (0.95, 1.00)	0.93	0.94	0.93	0.95	0.94
Internal test set						
Mask DWI	0.97 (0.93, 0.99)	0.83	0.97	0.89	0.97	0.90
Mask ADC620	0.98 (0.95, 1.00)	0.86	0.94	0.89	0.95	0.90
Mask ADC	0.97 (0.93, 0.99)	0.90	0.91	0.91	0.93	0.92
External test set						
Mask DWI	0.92 (0.85, 0.97)	0.82	0.85	0.84	0.76	0.79
Mask ADC620	0.91 (0.83, 0.98)	0.82	0.81	0.82	0.71	0.76
Mask ADC	0.88 (0.80, 0.96)	0.80	0.78	0.78	0.67	0.73

All variables are expressed as mean.

ADC, apparent diffusion coefficient; AUC, area under the curve; CI, confidence interval; DWI, diffusion-weighted imaging.

## Discussion

### Radiomics features improve the prognosis prediction of AACNLI

The AIS outcome can be evaluated by clinical and imaging information. Previous studies showed that the biomarkers of DWI and T2-FLAIR can be used for AIS prognosis prediction.^23-25^ As an effective supplement to radiological information, radiomics has been applied to the prognosis prediction of AIS. Quan *et al*.^8^ extracted 753 radiomics features from FLAIR and ADC images of 190 AIS patients and found 6 strongest radiomics features associated with poor prognosis. Tang *et al*.^26^ extracted 456 radiomics features from ADC and cerebral blood flow maps of 168 AIS patients and found R score higher in patients with favourable outcome. We extracted 293 radiomics features from FLAIR, DWI and ADC images and found that the radiomics features can improve the performance of predicting the AACNLI outcome.

The radiomics features can reflect subtle variations within the lesion not easily detected by the naked eye. We revealed moderately significant correlations between the prognosis and several histograms and texture features of AIS lesions on the MR images. In this study, high-order features were related to lesion’s shape, and second-order features were related to lesion’s heterogeneity. The first-order feature InterquartileRange_ADC reflected the severity of cytotoxic oedema.

### AIS segmentation affect efficacy of AACNLI outcome prediction

As for segmentation of acute ischaemic infarction on MRI, previous studies mainly focused on DWI high-signal lesion’s segmentation.^23,26^ However, restricted diffusion regions did not wholly transform into the final infarct. ADC is an objective measure of the diffusivity of water molecules in the tissue.^27^ We referred to the ADC threshold standard of ischaemic core^5,27^ and segmented the region with ADC < 620 × 10^−6^mm^2^/s in the DWI high-signal area, which can avoid overestimating the volume of ischaemic core due to low ADC caused by the normal grey matter nuclei with metal deposit.

The ischaemic core adjacent regions of ADC < 620 × 10^−6^mm²/s with DWI normal appearance were also segmented and analysed in this study. Blood supply to these regions is inadequate, especially white matter. Previous studies have shown that white matter is more tolerant to acute ischaemia and hypoxia than grey matter^28^ but much more sensitive to chronic ischaemia and hypoxia than grey matter.^29^ Irreversible axonal damage caused by demyelination may affect the prognosis of AACNLI. This study compared the value of these regional features in predicting AACNLI prognosis.

We calculated weighted average of the AUC score throughout the datasets and found that the RF model based on the mask ADC620 performed the best among the three mask types. It indicates that the features of ischaemic core and its adjacent region can predict prognosis more accurately than the traditional ischaemic core and DWI high-signal area, because the mask ADC620 contains more information about the ischaemic state of brain tissue. This segmentation method of ischaemic core–related regions can be further extended to evaluate therapeutic effect of intra-arterial mechanical thrombectomy in AIS and help screen more patients suitable for mechanical thrombectomy according to the prognostic prediction results.

### The optimal selection of FS-ML algorithm combinations improves the model’s performance

Previous studies often used limited algorithm to select and classify features. In this study, 12 FS algorithms and 9 ML algorithms were combined to construct the model, and the LASSO-RF algorithm combination was the most optimal algorithm combination according to the AUC results. This grid search selection method can fully evaluate the performance of various algorithm combinations, which lays a foundation for the construction of highly sensitive and specific prognosis prediction model.

LASSO regression algorithm is suitable for processing high-dimensional data with independent variables significantly larger than the sample size. Based on the sparsity hypothesis, continuous or discrete variables can be screened to reduce model complexity while fitting generalized linear models, thus avoiding overfitting.^30^ RF classification algorithm is to integrate multiple weak classifiers or decision trees into a forest, and the final result is processed by voting or averaging, so as to improve the accuracy of the constructed model and avoid overfitting. Therefore, the LASSO-RF algorithm combination can effectively process large samples with high-dimensional feature and accurately evaluate the weight of each feature in prognostic prediction.

### Clinical factors play more important roles in AACNLI outcome

In the AIS prognosis assessment, previous studies found that gender, age, admission NIHSS score and other clinical features were related to the prognosis.^31^ Imaging features, especially those derived from DWI and FLAIR images, were also associated with AIS prognosis.^32^ In this study, 7-d NIHSS_max_, SAP and admission GCS score were the top three important features in the prognostic prediction model.

According to the definition of early neurological deterioration, we collected the daily NIHSS score of patients within 7 days from the onset of AIS and calculated NIHSS_max_. Once early neurological deterioration appeared, most AIS patients would require longer neurological rehabilitation time, which may lead to higher incidence rate of poor prognosis.

SAP refers to new pneumonia in stroke patients with non-mechanical ventilation within 7 days from onset, and its pathogenesis is related to stroke-induced immunosuppression, swallowing dysfunction and down-migration of oral colonized flora.^33^ SAP may lead to high stroke mortality, prolonged hospitalization and poor neurological function recovery after discharge, which increases the difficulty of stroke treatment.^34,35^ In this study, SAP in the poor prognosis group accounted for 53.9%, much higher than that in the good prognosis group, indicating that SAP is an important factor leading to poor outcome. We can select AIS patients with SAP for more medical care, such as phlegm resolving and sputum drainage, oral care, anti-infective therapy and so on to improve the outcome.

GCS can reflect the consciousness, speaking and motor status of patients. Previous studies showed that the admission GCS score of patients who were functionally independent at discharge was higher than that of patients who were functionally dependent.^36^ In this study, the admission GCS score was lower in the poor prognosis group, indicating that low GCS score can reflect serious neurological function impairment.

### Limitations

Our study has several limitations. First of all, our sample size is limited, susceptible to selection bias. Second, the treatment of AACNLI cases is limited to traditional antiplatelet therapy, and thrombolysis and thrombectomy is not included in our study. Third, our study lacked hyperacute infarction cases. Finally, the differences of MRI equipment and sequence parameters in different stroke centres may affect the radiomics features of the lesion. In the future, we will build a large-sample, multicentre, standardized AACNLI clinical and imaging database, and carry out further research.

## Conclusions

Based on the clinical, radiological and radiomics features, LASSO-RF algorithm combination was most suitable for constructing AACNLI outcome prediction model. Seven-day NIHSS_max_, SAP and admission GCS scores were the three most important features in the model. Among the RF models of three mask types, mask ADC620 gained the best prognostic prediction performance, which helped to screen out high-risk patients with poor prognosis and identify the risk factors leading to poor outcome, providing an important basis for clinical precision treatment and improving the prognosis of AACNLI.

## Supplementary Material

fcae393_Supplementary_Data

## Data Availability

Data may be made available from the corresponding authors upon reasonable request.
