# Supplementary material for: Outcome prediction comparison of ischaemic areas’ radiomics in acute anterior circulation non-lacunar infarction
Source: Brain Commun. 2024 Nov 15;6(6):fcae393. doi: 10.1093/braincomms/fcae393 (PMC11580218; doi:10.1093/braincomms/fcae393)
Supplement: fcae393_Supplementary_Data [file fcae393_supplementary_data.docx]

**Supplementary Material**

# Supplementary Appendix 1. Conventional MRI variables of acute anterior-circulation non-lacuna infarction (AACNLI)

Fluid attenuated inversion recovery (FLAIR) images based Fazekas score’s range is 0-6, including periventricular white matter score (0-3) and deep white matter score (0-3), with 6 indicating confluent lesions and 0 indicating no lesions.^1^

Diffusion weighted imaging - Alberta Stroke Program Early CT Score’s (DWI-ASPECTS) range is 0-10, with 0 indicating diffuse infarct throughout the middle cerebral artery (MCA) territory and 10 indicating no lesion.^2^

Clot-burden score’s (CBS) range is 0-10, with 0 indicating unilateral anterior circulation aortic diffuse thrombosis and 10 indicating no thrombus.^3^

DWI-FLAIR mismatch is defined as a DWI lesion without corresponding marked FLAIR hyperintensity.^4^

FLAIR vessel hyperintensity (FVH) is defined as focal, tubular, or serpentine hyperintensities in the subarachnoid space relative to cerebrospinal fluid. FVH score’s range is 0-7, with 0 indicating no MCA collateral circulation and 7 indicating full MCA collateral circulation.^5^

DWI-FVH mismatch is defined as AIS lesion with FLAIR vascular hyperintensities observed beyond the boundaries of the cortical lesion on DWI.^6,7^

# Supplementary Appendix 1 References

1. Zhang P, Han R, Zhang A, *et al*. Association between Serum Amyloid A Level and White Matter Hyperintensity Burden: a Cross-Sectional Analysis in Patients with Acute Ischemic Stroke. Neurol Ther. 2023;12(1):161-175. doi: 10.1007/s40120-022-00415-y.
2. Terasawa Y, Shimomura R, Sato K, Himeno T, Inoue T, Kohriyama T. The efficacy and safety of alteplase treatment in patients with acute ischemic stroke with unknown time of onset: -Real world data. J Clin Neurosci. 2023;107:124-128. doi: 10.1016/j.jocn.2022.11.018.
3. Horsch AD, Dankbaar JW, Niesten JM, *et al*. Dutch Acute Stroke Study Investigators. Predictors of reperfusion in patients with acute ischemic stroke. AJNR Am J Neuroradiol. 2015;36(6):1056-62. doi: 10.3174/ajnr.A4283.
4. Annus Á, Gera FZ, Sztriha L, Klivényi P. DWI-FLAIR mismatch guided thrombolysis in patients without large-vessel occlusion: real-world data from a comprehensive stroke centre. Heliyon. 2022;8(12):e12069. doi: 10.1016/j.heliyon.2022.e12069.
5. Azizyan A, Sanossian N, Mogensen MA, Liebeskind DS. Fluid-attenuated inversion recovery vascular hyperintensities: an important imaging marker for cerebrovascular disease. AJNR Am J Neuroradiol. 2011;32(10):1771-5. doi: 10.3174/ajnr.A2265.
6. Xu XQ, Shen GC, Ma G, *et al*. Prognostic value of post-treatment fluid-attenuated inversion recovery vascular hyperintensity in ischemic stroke after endovascular thrombectomy. Eur Radiol. 2022;32(12):8067-8076. doi: 10.1007/s00330-022-08886-1.
7. Kaewumporn U, Songsaeng D, Kunapinun A, Chaisinanunkul N. FLAIR vascular hyperintensity-DWI mismatch and DWI-FLAIR mismatch ASPECTS for prediction of good outcome after recanalization in anterior circulation stroke; multicenter observational study. Eur J Radiol. 2023;163:110837. doi: 10.1016/j.ejrad.2023.110837.

# Supplementary Appendix 2. The radiological features of masks

This study measured and calculated the radiological features of mask as follows: total lesion volume (V), maximum lesion volume (V_max_), maximum lesion volume proportion (P) = V_max_ / V, the number of lesions (N), ADC mean of lesions (ADC_mean_), ADC standard deviation of lesions (ADC_SD_), and ADC coefficient of variation of lesions (ADC_CV_) = ADC_SD_ / ADC_mean_. We drew three round ROIs on the contralateral side of the lesions to calculate the averaged ADC (ADC_normal_), with the area of about 30mm^2^ - 60mm^2^. Then we calculated the relative ADC (rADC) = ADC_mean_ / ADC_normal_.

In addition, image gradient is used to reflect the rate of change in grayscale of two adjacent pixels in the x and y directions. This study calculated the mean of gradient (Grad_mean_), standard deviation of gradient (Grad_SD_) and coefficient of variation of gradient (Grad_CV_) of the AIS lesions on ADC images.

The calculation formula for lesion Grad_mean_ is as follows:

$$G=\frac{1}{M\times N}\sum_{i=1}^{M} \sum_{j=1}^{N} \sqrt{\frac{\left( \frac{\partial f}{\partial x} \right)^{2}+\left( \frac{\partial f}{\partial y} \right)^{2}}{2}}$$

Among them, M×N represents the lesion size, $\frac{\partial f}{\partial x}$ represents the horizontal gradient, and $\frac{\partial f}{\partial y}$ represents the vertical gradient.

The calculation formula for lesion Grad_SD_ is as follows:

$$\delta=\sqrt{\frac{1}{M\times N}\sum_{i=1}^{M} \sum_{j=1}^{N} {(P\left( i,j \right)-\mu)}^{2}}$$

Among them, P (*i*, *j*) represents the gradient values of row *i* and column *j*, *μ* represents the mean of gradient (Grad_mean_), *δ* represents the standard deviation of gradient (Grad_SD_).

Grad_CV_ = Grad_SD_/Grad_mean_. Grad_mean_ can reflect the degree of gray level fluctuation of each pixel within the lesion, while Grad_SD_ can reflect the difference in gray level fluctuation, both of which reflect the uniformity of the lesion. Grad_CV_ can reflect the degree of variation of grayscale fluctuations.

# Supplementary Appendix 3. The principal codes about radiomics feature extraction, feature selection and modeling

The principal codes about image preprocessing, radiomics feature extraction, feature selection (LASSO regression) and model construction (random forest) of this study were listed as follows.

import warnings

warnings.filterwarnings("ignore")

import os

import pickle

import numpy as np

import pandas as pd

import matplotlib.pyplot as plt

from scipy.stats import ttest_ind

from sklearn.preprocessing import MinMaxScaler, normalize

from sklearn.model_selection import LeaveOneOut, GridSearchCV

from sklearn import metrics

from sklearn.ensemble import RandomForestClassifier

from sklearn.metrics import confusion_matrix, plot_confusion_matrix, plot_roc_curve

# dcm2nii

filedir='C:/Users/Future/Desktop/Preprocessing/data1_dcm2nii'

from regist import *

for name in os.listdir(filedir):

dcm_path = os.path.join(filedir,name)

nii_path=os.path.join(filedir,name+'.nii')

dcm2nii(dcm_path, nii_path)

# make new file and move data (nii)

import shutil

from glob import glob

sourcefile = 'C:/Users/Future/Desktop/Preprocessing/data1_dcm2nii/'

destination = 'C:/Users/Future/Desktop/Preprocessing/data1_nii_remove/'

sourcefile_list = glob(os.path.join(sourcefile, "*.nii"))

for srcfile in sourcefile_list:

fpath,fname=os.path.split(srcfile)

os.makedirs(destination+fname[0:-4])

shutil.copy(srcfile,os.path.join(destination+fname[0:-4],'T1.nii') )

# N4biasCorrect

import SimpleITK as sitk

filedir='C:/Users/Future/Desktop/4Radiomics_RF_day4/Preprocessing/data2_n4'

for name in os.listdir(filedir):

imagePath=os.path.join(filedir,name,'T1.nii')

input_image = sitk.ReadImage(imagePath)

mask_image = sitk.OtsuThreshold(input_image,0,1,200)

input_image = sitk.Cast(input_image, sitk.sitkFloat32)

corrector = sitk.N4BiasFieldCorrectionImageFilter()

output_image = corrector.Execute(input_image,mask_image)

output_image = sitk.Cast(output_image, sitk.sitkInt16)

output_path=os.path.join(filedir,name,'image_N4.nii.gz')

mask_path=os.path.join(filedir,name,'image_mask.nii.gz')

sitk.WriteImage(output_image, output_path)

sitk.WriteImage(mask_image, mask_path)

# register

filedir='C:/Users/Future/Desktop/Preprocessing/data3_register'

for name in os.listdir(filedir):

listdata = os.listdir(os.path.join(filedir, name))

fixed_image = os.path.join(filedir,name,listdata[0])

moving_image = os.path.join(filedir,name,listdata[1])

output_dir = os.path.join(filedir,name,'w'+listdata[1])

regist_image(fixed_image, moving_image, output_dir)

# feature_extract

from radiomics import featureextractor

feature_all=pd.DataFrame();

filedir= r'C:/Users/Future/Desktop/Preprocessing/data4_Feature_Extract/Group'

for name in os.listdir(filedir):

params='C:/Users/Future/Desktop/Preprocessing/data4_Feature_Extract/MR_3mm.yaml';

extractor=featureextractor.RadiomicsFeatureExtractor(params)

feature_1=extractor.execute(os.path.join(filedir,name,'image.nii'),

os.path.join(filedir,name,'roi.nii'))

featureA=pd.DataFrame([feature_1])

feature_1=np.transpose(featureA)

feature_all=pd.concat([feature_all,feature_1],axis=1)

feature_all=feature_all.T

fea=feature_all.iloc[:,23:]

fea.to_csv(os.path.join('reader1.csv'))

# import feature set and labels

import csv

csv_data = []

with open('data1.csv') as f:

csv_content = csv.reader(f)

for row in csv_content:

csv_data.append(row)

csv_data = csv_data[1:]

csv_data = np.array(csv_data)

csv_data = csv_data.astype('float64')

all_data = csv_data[:,1:]

labels = csv_data[:,0]

# scaler

scaler = MinMaxScaler(feature_range=(-1,1))

scaler.fit(train_data)

train_data = scaler.transform(train_data)

test_data = scaler.transform(test_data)

# feature_selection (LASSO)

from sklearn.preprocessing import normalize

from sklearn.feature_selection import SelectFromModel

selector = SelectFromModel(regr2, prefit=True)

all_data_selected = selector.transform(all_data)

LASSO_coef = regr2.coef_

all_data_selected = np.squeeze(all_data[:,np.where(LASSO_coef != 0)])

alphas = np.power(2.0, np.arange(-5,6))

LASSO_CV_model = linear_model.LassoCV(alphas = alphas, cv=10)

LASSO_CV_model.fit(all_data, labels)

selector = SelectFromModel(LASSO_CV_model, prefit=True)

all_data_selected = selector.transform(all_data)

LASSO_CV_model.alpha_

# tunning hyper-parameters

tree_num = (10,100,1000)

feature_num = (10,30)

params = {'n_estimators': tree_num, 'max_features': feature_num}

forest = RandomForestClassifier()

clf = GridSearchCV(forest, params)

clf.fit(train_data, train_label)

best_params = clf.best_params_

# Random forest model

foresti = RandomForestClassifier(**best_params)

foresti.fit(train_data, train_label)

predicted_label = foresti.predict(test_data)

p_labels.append(predicted_label)

predicted_proba = foresti.predict_proba(test_data)

probas.append(predicted_proba)

# weight

w = foresti.feature_importances_

weights.append(w)

print('CV %d done...' % test_idx)

p_labels = np.squeeze(p_labels)

cmat = confusion_matrix(labels, p_labels) # confusion matrix

TN, FP, FN, TP = cmat.ravel()

accuracy = (TP + TN) /(TP + FN + FP + TN)

precision = TP /(TP + FP)

sensitivity = TP / (TP + FN)

specificity = TN /(TN + FP)

F1 = 2 * precision * sensitivity / (precision + sensitivity)

#%% Test Set

# keep the consensus features

all_data_selected = all_data[:, T_idx_sorted[:filtered_feature_num]]

# train with best params & probability output

forest = RandomForestClassifier(n_estimators=172, max_features=30)

forest.fit(all_data_selected, labels)

# open the Test set

f = open('data1.dat', 'rb')

val_dataset = pickle.load(f)

f.close()

val_data = val_dataset['all_data']

val_labels = val_dataset['labels']

val_data_selected = val_data[:, T_idx_sorted[:filtered_feature_num]]

# predict on the validation set

val_p_labels = forest.predict(val_data_selected)

val_acc_final = np.mean(val_labels == val_p_labels)

# ROC, AUC

from sklearn.metrics import roc_curve, auc, roc_auc_score

plt.figure(figsize=(20, 20), dpi=600)

roc_auc = roc_auc_score(labels, p_labels)

fpr, tpr, thds = roc_curve(y_true=labels, y_score=p_labels, pos_label=1)

plt.plot(fpr, tpr, lw=3, label='{} (AUC={:.3f})'.format('RF', roc_auc),color = 'black')

plt.plot([0, 1], [0, 1], '--', lw=3, color = 'grey')

plt.axis('square')

plt.xlim([0, 1])

plt.ylim([0, 1])

plt.xlabel('False Positive Rate',fontsize=20)

plt.ylabel('True Positive Rate',fontsize=20)

plt.title('ROC Curve',fontsize=25)

plt.legend(loc='lower right',fontsize=20)

# Calibration Curve

from sklearn.calibration import calibration_curve

probas_arr = np.squeeze(np.array(probas))

y_prob = probas_arr[:,1]

prob_true, prob_pred = calibration_curve(labels, y_prob, n_bins=10)

plt.figure(figsize=(12, 6),dpi=600)

plt.plot(prob_pred, prob_true, marker='o')

plt.plot([0, 1], [0, 1], linestyle='--')

plt.xlabel('Average Predicted Value')

plt.ylabel('Proportion of Positive Records')

# Decision Curve Analysis

from Decision_Curve_Analysis import plot_decision_curves2

plot_decision_curves2([foresti], ['RF'], val_data_selected, val_labels, 0, 1, 0.02, -2, 0.7)

# Supplementary Table 1. Scan protocols of MRI

| **MR Equipment and**  **Sequence Name** | **TR**  **(ms)** | **TE**  **(ms)** | **Slice Thickness**  **(mm)** | **Spacing between slices**  **(mm)** | **FOV**  **(mm)** | **Acquisition Matrix** | **NEX** |
| --- | --- | --- | --- | --- | --- | --- | --- |
| **SIEMENS Verio** |  |  |  |  |  |  |  |
| Axial T1WI | 1530 | 9 | 5 | 6 | 230×230 | 320×240 | 1 |
| Axial T2WI | 4210 | 96 | 5 | 6 | 230×230 | 320×245 | 1 |
| Axial T2-FLAIR fat-suppression | 5500 | 94 | 5 | 6 | 230×230 | 256×151 | 1 |
| Axial T2-GRE | 550 | 20 | 5 | 6 | 208×230 | 256×186 | 1 |
| Axial DWI | 5400 | 94 | 5 | 6 | 230×230 | 162×162 | 1 |
| **United Imaging uMR 770** |  |  |  |  |  |  |  |
| Axial T1WI | 2267 | 13 | 5 | 6 | 230×230 | 352×264 | 1 |
| Axial T2WI | 4248 | 118 | 5 | 6 | 230×230 | 320×320 | 1 |
| Axial T2-FLAIR fat-suppression | 8100 | 105 | 5 | 6 | 230×230 | 304×228 | 1 |
| Axial T2-GRE | 286 | 8 | 5 | 6 | 184×230 | 280×160 | 1 |
| Axial DWI | 2196 | 79 | 5 | 6 | 230×230 | 168×168 | 1 |
| **Philips Ingenia** |  |  |  |  |  |  |  |
| Axial T1WI | 1800 | 20 | 5 | 6 | 230×230 | 288×209 | 1 |
| Axial T2WI | 2500 | 80 | 5 | 6 | 230×230 | 288×240 | 1 |
| Axial T2-FLAIR fat-suppression | 9000 | 120 | 5 | 6 | 230×230 | 352×173 | 1 |
| Axial T2-GRE | 562 | 16 | 5 | 6 | 184×230 | 328×262 | 1 |
| Axial DWI | 2604 | 96 | 5 | 6 | 230×230 | 152×122 | 1 |

DWI, diffusion weighted imaging; FLAIR, fluid attenuated inversion recovery; FOV, field of view; NEX, number of excitations; T1WI, T1 weighted imaging; T2WI, T2 weighted imaging; T2-GRE, T2 weighted gradient-echo imaging; TE, echo time; TR, repetition time.

The DWI scanning used Echo Planner Imaging (EPI) sequence, with an echo spacing of 0.75 ms and b values of 0 and 1000 s/mm^2^, to automatically generate ADC images.

# Supplementary Table 2. Clinical and radiological features of AACNLI patients in the training set and internal test set

| **Features** | **Training set (*n* = 172)** | **Internal test set (*n* = 75)** | ***p* value** |
| --- | --- | --- | --- |
| **Demography** |  |  |  |
| Female | 72 (41.8%) | 32 (42.7%) | 0.906 |
| Age | 72 (63, 84) | 71 (63, 80) | 0.516 |
| **History** |  |  |  |
| Smoking | 81 (47.1%) | 32 (42.7%) | 0.521 |
| Alcohol consumption | 47 (27.3%) | 19 (25.3%) | 0.745 |
| Diabetes | 53 (30.8%) | 27 (36.0%) | 0.423 |
| Myocardial infarction | 5 (2.9%) | 1 (1.3%) | 0.772 |
| Coronary atherosclerosis | 35 (20.3%) | 16 (21.3%) | 0.860 |
| Atrial fibrillation | 40 (23.3%) | 22 (29.3%) | 0.311 |
| Hypertension | 122 (70.9%) | 52 (69.3%) | 0.800 |
| Stroke | 50 (29.1%) | 16 (21.3%) | 0.206 |
| Heart Failure | 29 (16.9%) | 14 (18.7%) | 0.731 |
| Hyperlipidemia | 18 (10.5%) | 10 (13.3%) | 0.513 |
| Hyperhomocysteinemia | 3 (1.7%) | 2 (2.7%) | >0.999 |
| **Secondary disease** |  |  |  |
| Cognitive impairment | 6 (3.5%) | 1 (1.3%) | 0.602 |
| Epilepsy | 5 (2.9%) | 1 (1.3%) | 0.772 |
| SAP | 50 (29.1%) | 20 (26.7%) | 0.700 |
| **Blood pressure on admission** |  |  |  |
| SBP (mmHg) | 149.91 (21.20) | 148.15 (22.41) | 0.556 |
| DBP (mmHg) | 82 (77, 90) | 80 (77, 90) | 0.657 |
| **TOAST type** |  |  | 0.222 |
| Large-artery atherosclerosis | 119 (69.2%) | 56 (74.7%) |  |
| Cardio embolism | 32 (18.6%) | 16 (21.3%) |  |
| Small-artery occlusion | 17 (9.9%) | 2 (2.7%) |  |
| Other determined etiology | 4 (2.3%) | 1 (1.3%) |  |
| **OCSP type** |  |  | >0.999 |
| TACI | 31 (18.0%) | 13 (17.3%) |  |
| PACI | 138 (80.2%) | 61 (81.3%) |  |
| LACI | 3 (1.7%) | 1 (1.3%) |  |
| **Neurological scale score** |  |  |  |
| Admission NIHSS | 6 (3, 13) | 6 (3, 14) | 0.843 |
| Admission GCS | 15 (13, 15) | 15 (13, 15) | 0.904 |
| 7d NIHSS_max_ | 7 (4, 15) | 10 (4, 15) | 0.470 |
| **Laboratory test** |  |  |  |
| CRP (mg/L) | 4.18 (1.69, 10.32) | 5.66 (1.60, 12.14) | 0.576 |
| PLT (×10^9^/L) | 199 (166, 243) | 201 (163, 242) | 0.942 |
| PT (s) | 11.2 (10.7, 11.8) | 11.1 (10.7, 11.8) | 0.675 |
| Fibrinogen (g/L) | 2.88 (2.50, 3.36) | 2.98 (2.54, 3.52) | 0.706 |
| D-dimer (mg/L) | 0.58 (0.32, 1.26) | 0.55 (0.32, 0.95) | 0.482 |
| Serum troponin I (ng/mL) | 0.010 (0.009, 0.023) | 0.010 (0.008, 0.015) | 0.082 |
| Blood sugar (mmol/L) | 6.63 (5.53, 8.31) | 6.43 (5.67, 9.20) | 0.961 |
| Triglyceride (mmol/L) | 1.21 (0.94, 1.63) | 1.22 (0.94, 1.75) | 0.453 |
| Plasma BNP (pg/mL) | 90.7 (53.2, 281.3) | 84.0 (47.6, 246.5) | 0.513 |
| **Follow-up** |  |  |  |
| 3-month mRS | 2 (1, 4) | 3 (1, 4) | 0.222 |
| **MRI features** |  |  |  |
| Interval from onset to MRI (h) | 33 (21, 50) | 38 (23, 53) | 0.205 |
| CBS | 10 (7, 10) | 9 (6, 10) | 0.252 |
| HT | 24 (14.0%) | 10 (13.3%) | 0.897 |
| DWI-ASPECTS | 6 (4, 8) | 6 (4, 8) | 0.219 |
| Contralateral brain ADC (×10^-6^mm^2^/s) | 777.80 (52.61) | 785.13 (49.34) | 0.306 |
| DWI-FLAIR mismatch | 164 (95.3%) | 72 (96.0%) | 0.820 |
| DWI-FVH mismatch | 56 (32.6%) | 30 (40.0%) | 0.259 |
| FVH score | 2 (1, 4) | 3 (1, 4) | 0.146 |
| Fazekas score | 3 (2, 3) | 2 (1, 3) | 0.145 |

All categorical variables are expressed as n (%) and continuous variables as median (IQR) or mean (SD). AACNLI, acute anterior-circulation non-lacuna infarction; ADC, apparent diffusion coefficient; ASPECTS, Alberta Stroke Program Early CT Score; BNP, brain natriuretic peptide; CBS, clot burden score; CRP, C-reactive protein; DBP, diastolic blood pressure; DWI, diffusion weighted imaging; FLAIR, fluid attenuated inversion recovery; FVH, FLAIR vascular hyperintensity; GCS, Glasgow coma scale; HT, hemorrhage transformation; IQR, interquartile range; LACI, lacunar infarction; mRS, modified Rankin scale; NIHSS, National Institute of Health Stroke Scale; OCSP, Oxfordshire Community Stroke Project; PACI, partial anterior circulation infarction; PLT, platelet count; PT, prothrombin time; SAP, stroke-associated pneumonia; SBP, systolic blood pressure; SD, standard deviation; TACI, total anterior circulation infarction; TOAST, Trial of Org10172 in Acute Stroke Treatment.

# Supplementary Table 3. Clinical features of AACNLI patients with good and poor outcome (external test set)

| **Features** | **Good Outcome (n = 80)** | **Poor Outcome (n = 45)** | ***p* value** |
| --- | --- | --- | --- |
| **Demography** |  |  |  |
| Female | 28 (22.4%) | 24 (19.2%) | 0.046 |
| Age | 70 (62, 76) | 77 (65, 89) | 0.011 |
| **History** |  |  |  |
| Smoking | 33 (26.4%) | 16 (12.8%) | 0.531 |
| Alcohol consumption | 16 (12.8%) | 9 (7.2%) | >0.999 |
| Diabetes | 26 (20.8%) | 22 (17.6%) | 0.071 |
| Myocardial infarction | 3 (2.4%) | 2 (1.6%) | >0.999 |
| Coronary atherosclerosis | 19 (15.2%) | 15 (12.0%) | 0.248 |
| Atrial fibrillation | 16 (12.8%) | 11 (8.8%) | 0.562 |
| Hypertension | 54 (43.2%) | 38 (30.4%) | 0.039 |
| Stroke | 23 (18.4%) | 15 (12.0%) | 0.593 |
| Heart Failure | 6 (4.8%) | 4 (3.2%) | >0.999 |
| Hyperlipidemia | 12 (9.6%) | 12 (9.6%) | 0.112 |
| Hyperhomocysteinemia | 3 (2.4%) | 4 (3.2%) | 0.427 |
| **Secondary disease** |  |  |  |
| Cognitive impairment | 5 (4.0%) | 3 (2.4%) | >0.999 |
| Epilepsy | 2 (1.6%) | 1 (0.8%) | >0.999 |
| SAP | 5 (4.0%) | 17 (13.6%) | <0.001 |
| **Blood pressure on admission** |  |  |  |
| SBP (mmHg) | 146.09 (21.65) | 152.42 (23.32) | 0.129 |
| DBP (mmHg) | 81.00 (75.50, 93.00) | 81.00 (76.50, 94.00) | 0.709 |
| **TOAST type** |  |  | 0.205 |
| Large-artery atherosclerosis | 45 (36.0%) | 30 (24.0%) |  |
| Cardio embolism | 10 (8.0%) | 8 (6.4%) |  |
| Small-artery occlusion | 24 (19.2%) | 6 (4.8%) |  |
| Other determined etiology | 1 (0.8%) | 1 (0.8%) |  |
| **OCSP type** |  |  | 0.005 |
| TACI | 1 (0.8%) | 7 (5.6%) |  |
| PACI | 74 (59.2%) | 37 (29.6%) |  |
| LACI | 5 (4.0%) | 1 (0.8%) |  |
| **Neurological scale score** |  |  |  |
| Admission NIHSS | 3 (2, 4) | 9 (6, 14) | <0.001 |
| Admission GCS | 15 (15, 15) | 14 (11, 15) | <0.001 |
| 7d NIHSS_max_ | 3 (2, 5) | 10 (8, 16) | <0.001 |
| **Laboratory test** |  |  |  |
| CRP (mg/L) | 4.18 (1.69, 10.32) | 5.66 (1.60, 12.14) | 0.576 |
| PLT (×10^9^/L) | 199 (166, 243) | 201 (163, 242) | 0.942 |
| PT (s) | 11.2 (10.7, 11.8) | 11.1 (10.7, 11.8) | 0.675 |
| Fibrinogen (g/L) | 2.88 (2.50, 3.36) | 2.98 (2.54, 3.52) | 0.706 |
| D-dimer (mg/L) | 0.58 (0.32, 1.26) | 0.55 (0.32, 0.95) | 0.482 |
| Serum troponin I (ng/mL) | 0.010 (0.009, 0.023) | 0.010 (0.008, 0.015) | 0.082 |
| Blood sugar (mmol/L) | 6.63 (5.53, 8.31) | 6.43 (5.67, 9.20) | 0.961 |
| Triglyceride (mmol/L) | 1.21 (0.94, 1.63) | 1.22 (0.94, 1.75) | 0.453 |
| Plasma BNP (pg/mL) | 90.7 (53.2, 281.3) | 84.0 (47.6, 246.5) | 0.513 |
| **Follow-up** |  |  |  |
| 3-month mRS | 1 (1, 1) | 4 (3, 5) | <0.001 |

All categorical variables are expressed as n (%) and continuous variables as median (IQR) or mean (SD). AACNLI, acute anterior-circulation non-lacuna infarction; BNP, brain natriuretic peptide; CRP, C-reactive protein; DBP, diastolic blood pressure; GCS, Glasgow coma scale; IQR, interquartile range; LACI, lacunar infarction; mRS, modified Rankin scale; NIHSS, National Institute of Health Stroke Scale; OCSP, Oxfordshire Community Stroke Project; PACI, partial anterior circulation infarction; PLT, platelet count; PT, prothrombin time; SAP, stroke-associated pneumonia; SBP, systolic blood pressure; SD, standard deviation; TACI, total anterior circulation infarction; TOAST, Trial of Org10172 in Acute Stroke Treatment.

# Supplementary Table 4. Radiological features of AACNLI patients with good and poor outcome (external test set)

| **Features** | **Good Outcome (n = 80)** | **Poor Outcome (n = 45)** | **p value** |
| --- | --- | --- | --- |
| Interval from onset to MRI (h) | 36 (22, 52) | 33 (20, 51.5) | 0.344 |
| CBS | 10 (10, 10) | 10 (9, 10) | <0.001 |
| HT | 2 (1.6%) | 4 (3.2%) | 0.243 |
| DWI-ASPECTS | 8 (7, 9) | 8 (5, 9) | 0.005 |
| Contralateral brain ADC  (×10^-6^mm^2^/s) | 773 (60) | 796 (62) | 0.044 |
| DWI-FLAIR mismatch | 79 (63.2%) | 42 (33.6%) | 0.262 |
| DWI-FVH mismatch | 8 (6.4%) | 13 (10.4%) | 0.007 |
| FVH score | 0 (0, 0) | 0 (0, 2) | 0.031 |
| Fazekas score | 2 (2, 3) | 3 (2, 4) | 0.052 |
| **Mask DWI features** |  |  |  |
| V1 (mL) | 6.35 (2.91, 13.05) | 12.86 (5.15, 70.85) | 0.001 |
| V1max (mL) | 4.48 (1.74, 8.39) | 11.23 (3.02, 63.49) | <0.001 |
| P1 | 0.89 (0.56, 1.00) | 0.98 (0.88, 1.00) | 0.093 |
| N1 | 4 (1, 12) | 5 (2, 12) | 0.444 |
| ADC1mean (×10^-6^mm^2^/s) | 570 (529, 676) | 533 (487, 606) | 0.015 |
| ADC1SD (×10^-6^mm^2^/s) | 162 (137, 200) | 175 (147, 206) | 0.254 |
| ADC1CV | 0.29 (0.23, 0.34) | 0.31 (0.28, 0.37) | 0.013 |
| Grad1mean | 2859 (2213, 4103) | 2822 (2351, 3407) | 0.777 |
| Grad1SD | 1645 (1257, 2625) | 1696 (1301, 2032) | 0.992 |
| Grad1CV | 0.56 (0.11) | 0.59 (0.13) | 0.216 |
| rADC1 | 0.75 (0.68, 0.86) | 0.70 (0.62, 0.75) | 0.001 |
| **Mask ADC620 features** |  |  |  |
| V2 (mL) | 3.46 (1.81, 8.53) | 11.17 (3.62, 51.19) | <0.001 |
| V2max (mL) | 2.46 (1.12, 6.64) | 9.12 (2.73, 49.54) | <0.001 |
| P2 | 0.89 (0.64, 0.99) | 0.98 (0.89, 1.00) | 0.011 |
| N2 | 6 (2, 15) | 8 (2, 20) | 0.258 |
| ADC2mean (×10^-6^mm^2^/s) | 494 (38) | 470 (35) | <0.001 |
| ADC2SD (×10^-6^mm^2^/s) | 77 (19) | 90 (17) | <0.001 |
| ADC2CV | 0.15 (0.13, 0.20) | 0.18 (0.16, 0.23) | <0.001 |
| Grad2mean | 7501 (5886, 9113) | 7952 (6912, 10127) | 0.123 |
| Grad2SD | 6035 (2162) | 6699 (1884) | 0.087 |
| Grad2CV | 0.78 (0.66, 0.90) | 0.81 (0.67, 0.96) | 0.255 |
| rADC2 | 0.64 (0.07) | 0.59 (0.06) | <0.001 |
| **Mask ADC features** |  |  |  |
| V3 (mL) | 2.99 (1.63, 7.27) | 9.49 (3.06, 47.61) | <0.001 |
| V3max (mL) | 2.12 (0.91, 4.76) | 8.71 (2.44, 46.09) | <0.001 |
| P3 | 0.88 (0.60, 0.99) | 0.97 (0.85, 1.00) | 0.032 |
| N3 | 6 (2, 16) | 8 (2, 22) | 0.350 |
| ADC3mean (×10^-6^mm^2^/s) | 488 (42) | 462 (40) | 0.001 |
| ADC3SD (×10^-6^mm^2^/s) | 77 (19) | 89 (16) | 0.001 |
| ADC3CV | 0.16 (0.13, 0.20) | 0.18 (0.16, 0.23) | 0.001 |
| Grad3mean | 7505 (5864, 9001) | 7578 (6766, 10271) | 0.137 |
| Grad3SD | 6206 (4457, 7439) | 6919 (5702, 7923) | 0.085 |
| Grad3CV | 0.77 (0.18) | 0.82 (0.19) | 0.210 |
| rADC3 | 0.63 (0.07) | 0.58 (0.07) | <0.001 |

All categorical variables are expressed as n (%) and continuous variables as median (IQR) or mean (SD). AACNLI, acute anterior-circulation non-lacuna infarction; ADC, apparent diffusion coefficient; ASPECTS, Alberta Stroke Program Early CT Score; CBS, clot burden score; CV, coefficient of variation; DWI, diffusion weighted imaging; FLAIR, fluid attenuated inversion recovery; FVH, FLAIR vascular hyperintensity; Grad, gradient; HT, hemorrhage transformation; IQR, interquartile range; N, number of the lesions; P, proportion (Vmax/V); rADC, relative apparent diffusion coefficient; SD, standard deviation. V, total volume of the lesions; Vmax, volume of the largest lesion.

# Supplementary Table 5. Radiomics Feature Selection Algorithm

| **Number** | **Abbreviation** | **Full Name** |
| --- | --- | --- |
| 1 | CIFE | Common and Individual Feature Extraction |
| 2 | CMIM | Conditional Mutual Information Maximization |
| 3 | DISR | Dental Image Segmentation and Retrieval |
| 4 | Fast ICA | Fast Independent Component Analysis |
| 5 | ICAP | Interaction Capping |
| 6 | JMI | Joint Mutual Information |
| 7 | LASSO | Least Absolute Shrinkage and Selection Operator |
| 8 | MIM | Mutual Information Maximization |
| 9 | NMF | Non-negative Matrix Factorization |
| 10 | None | All features without any selection |
| 11 | PCA | Principal Component Analysis |
| 12 | Truncated SVD | Truncated Singular Value Decomposition |

# Supplementary Table 6. Machine Learning Classification Algorithm

| **Number** | **Abbreviation** | **Full Name** |
| --- | --- | --- |
| 1 | Adaboost | Adaptive Boosting |
| 2 | DET | Deep Extremely Randomized Trees |
| 3 | EXT | Extremely Randomized Trees |
| 4 | KNN | K-Nearest Neighbor |
| 5 | LR | Logistic Regression |
| 6 | MLP | Multi-layer Perceptron |
| 7 | RF | Random Forest |
| 8 | SVM | Support Vector Machine |
| 9 | Xgboost | Extreme Gradient Boosting |

# Supplementary Table 7. The AUC comparison of clinical + radiological + radiomics model with clinical + radiological model in the three mask types used by training data

| **Radiomics model** | |  | **Non-radiomics model** | | ***Z*** | ***p* value** |
| --- | --- | --- | --- | --- | --- | --- |
| **FS-ML algorithm combination** | **AUC** |  | **ML algorithm** | **AUC** |  |  |
| **Mask DWI** |  |  |  |  |  |  |
| ICAP-Adaboost | 0.99 (0.01) |  | Adaboost | 0.97 (0.01) | 0.940 | 0.347 |
| ICAP-DET | 0.90 (0.03) |  | DET | 0.90 (0.03) | 0.162 | 0.872 |
| ICAP-EXT | 0.98 (0.01) |  | EXT | 0.97 (0.01) | 0.768 | 0.442 |
| LASSO-KNN | 0.97 (0.01) |  | KNN | 0.86 (0.04) | 2.724 | 0.006 |
| LASSO-LR | 0.97 (0.01) |  | LR | 0.96 (0.01) | 0.610 | 0.542 |
| LASSO-MLP | 0.97 (0.01) |  | MLP | 0.95 (0.02) | 0.782 | 0.434 |
| LASSO-RF | 0.98 (0.01) |  | RF | 0.96 (0.01) | 1.097 | 0.272 |
| DISR-SVM | 0.98 (0.01) |  | SVM | 0.96 (0.01) | 1.280 | 0.200 |
| ICAP-Xgboost | 0.97 (0.01) |  | Xgboost | 0.95 (0.02) | 0.673 | 0.501 |
| **Mask ADC620** |  |  |  |  |  |  |
| ICAP-Adaboost | 0.99 (0.01) |  | Adaboost | 0.96 (0.01) | 1.562 | 0.118 |
| ICAP-DET | 0.90 (0.03) |  | DET | 0.90 (0.03) | 0.138 | 0.890 |
| ICAP-EXT | 0.99 (0.01) |  | EXT | 0.97 (0.01) | 0.832 | 0.405 |
| DISR-KNN | 0.96 (0.01) |  | KNN | 0.87 (0.04) | 2.100 | 0.036 |
| DISR-LR | 0.97 (0.01) |  | LR | 0.97 (0.01) | 0.157 | 0.875 |
| LASSO-MLP | 0.97 (0.01) |  | MLP | 0.95 (0.02) | 0.737 | 0.461 |
| LASSO-RF | 0.99 (0.01) |  | RF | 0.98 (0.01) | 0.937 | 0.349 |
| DISR-SVM | 0.98 (0.01) |  | SVM | 0.95 (0.02) | 1.360 | 0.174 |
| ICAP-Xgboost | 0.98 (0.01) |  | Xgboost | 0.95 (0.02) | 1.280 | 0.201 |
| **Mask ADC** |  |  |  |  |  |  |
| LASSO-Adaboost | 0.99 (0.01) |  | Adaboost | 0.98 (0.01) | 1.308 | 0.191 |
| ICAP-DET | 0.90 (0.03) |  | DET | 0.90 (0.03) | 0.070 | 0.944 |
| ICAP-EXT | 0.99 (0.01) |  | EXT | 0.99 (0.01) | 0.223 | 0.824 |
| LASSO-KNN | 0.96 (0.01) |  | KNN | 0.88 (0.04) | 1.626 | 0.104 |
| DISR-LR | 0.97 (0.01) |  | LR | 0.97 (0.01) | 0.235 | 0.814 |
| LASSO-MLP | 0.97 (0.01) |  | MLP | 0.94 (0.02) | 1.134 | 0.257 |
| LASSO-RF | 0.98 (0.01) |  | RF | 0.97 (0.01) | 0.854 | 0.393 |
| DISR-SVM | 0.97 (0.01) |  | SVM | 0.96 (0.01) | 1.067 | 0.286 |
| ICAP-Xgboost | 0.98 (0.01) |  | Xgboost | 0.92 (0.03) | 1.878 | 0.060 |

AUCs are expressed as mean (SD). ADC, apparent diffusion coefficient; AUC, area under the curve; DWI, diffusion weighted imaging; FS, feature selection; ML, machine learning; SD, standard deviation.

# Supplementary Table 8. MRI Radiomics Features of mask DWI

| **Sequence** | **Feature Type** | **Feature Name** |
| --- | --- | --- |
| ADC | High order | original_shape_Maximum2DDiameterRow_ADC |
| ADC | First order | original_firstorder_InterquartileRange_ADC |
| ADC | First order | original_firstorder_Minimum_ADC |
| ADC | Second order | original_glrlm_ShortRunLowGrayLevelEmphasis_ADC |
| ADC | Second order | original_glszm_GrayLevelNonUniformityNormalized_ADC |
| ADC | Second order | original_glszm_SizeZoneNonUniformityNormalized_ADC |
| ADC | Second order | original_glszm_SmallAreaHighGrayLevelEmphasis_ADC |
| ADC | Second order | original_glszm_ZoneVariance_ADC |
| ADC | Second order | original_ngtdm_Busyness_ADC |
| ADC | Second order | original_ngtdm_Contrast_ADC |
| ADC | Second order | original_ngtdm_Strength_ADC |
| DWI | First order | original_firstorder_10Percentile_DWI |
| DWI | Second order | original_glcm_Imc1_DWI |
| DWI | Second order | original_glszm_HighGrayLevelZoneEmphasis_DWI |
| DWI | Second order | original_glszm_SizeZoneNonUniformity_DWI |
| DWI | Second order | original_ngtdm_Complexity_DWI |
| FLAIR | First order | original_firstorder_10Percentile_FLAIR |
| FLAIR | First order | original_firstorder_Median_FLAIR |
| FLAIR | First order | original_firstorder_Range_FLAIR |
| FLAIR | First order | original_firstorder_RobustMeanAbsoluteDeviation_FLAIR |
| FLAIR | Second order | original_glcm_ClusterShade_FLAIR |
| FLAIR | Second order | original_glcm_Correlation_FLAIR |
| FLAIR | Second order | original_glrlm_GrayLevelNonUniformity_FLAIR |
| FLAIR | Second order | original_glrlm_LongRunLowGrayLevelEmphasis_FLAIR |
| FLAIR | Second order | original_glszm_SmallAreaEmphasis_FLAIR |
| FLAIR | Second order | original_glszm_SmallAreaHighGrayLevelEmphasis_FLAIR |
| FLAIR | Second order | original_ngtdm_Busyness_FLAIR |

ADC, apparent diffusion coefficient; DWI, diffusion weighted imaging; FLAIR, fluid attenuated inversion recovery; glcm, gray-level cooccurrence matrix; gldm, gray-level difference matrix; glrlm, gray-level run-length matrix; glszm, gray-level size zone matrix; ngtdm, neighbourhood gray-tone difference matrix.

# Supplementary Table 9. MRI Radiomics Features of mask ADC620

| **Sequence** | **Feature Type** | **Feature Name** |
| --- | --- | --- |
| ADC | High order | original_shape_Elongation_ADC |
| ADC | High order | original_shape_Maximum2DDiameterColumn_ADC |
| ADC | High order | original_shape_Maximum2DDiameterRow_ADC |
| ADC | First order | original_firstorder_Energy_ADC |
| ADC | First order | original_firstorder_InterquartileRange_ADC |
| ADC | Second order | original_glcm_ClusterProminence_ADC |
| ADC | Second order | original_glcm_Imc1_ADC |
| ADC | Second order | original_gldm_SmallDependenceHighGrayLevelEmphasis_ADC |
| ADC | Second order | original_glrlm_ShortRunEmphasis_ADC |
| ADC | Second order | original_glszm_SizeZoneNonUniformity_ADC |
| ADC | Second order | original_glszm_SizeZoneNonUniformityNormalized_ADC |
| ADC | Second order | original_glszm_SmallAreaEmphasis_ADC |
| ADC | Second order | original_ngtdm_Strength_ADC |
| DWI | Second order | original_glrlm_GrayLevelNonUniformityNormalized_DWI |
| DWI | Second order | original_glrlm_GrayLevelVariance_DWI |
| DWI | Second order | original_glszm_GrayLevelNonUniformityNormalized_DWI |
| DWI | Second order | original_glszm_SizeZoneNonUniformity_DWI |
| DWI | Second order | original_glszm_SmallAreaLowGrayLevelEmphasis_DWI |
| DWI | Second order | original_ngtdm_Complexity_DWI |
| DWI | Second order | original_ngtdm_Contrast_DWI |
| DWI | Second order | original_ngtdm_Strength_DWI |
| FLAIR | First order | original_firstorder_10Percentile_FLAIR |
| FLAIR | First order | original_firstorder_Entropy_FLAIR |
| FLAIR | First order | original_firstorder_Median_FLAIR |
| FLAIR | Second order | original_glcm_Contrast_FLAIR |
| FLAIR | Second order | original_gldm_LargeDependenceLowGrayLevelEmphasis_FLAIR |
| FLAIR | Second order | original_glszm_HighGrayLevelZoneEmphasis_FLAIR |
| FLAIR | Second order | original_ngtdm_Strength_FLAIR |

ADC, apparent diffusion coefficient; DWI, diffusion weighted imaging; FLAIR, fluid attenuated inversion recovery; glcm, gray-level cooccurrence matrix; gldm, gray-level difference matrix; glrlm, gray-level run-length matrix; glszm, gray-level size zone matrix; ngtdm, neighbourhood gray-tone difference matrix.

# Supplementary Table 10. MRI Radiomics Features of mask ADC

| **Sequence** | **Feature Type** | **Feature Name** |
| --- | --- | --- |
| ADC | High order | original_shape_Maximum2DDiameterColumn_ADC |
| ADC | High order | original_shape_Maximum2DDiameterRow_ADC |
| ADC | High order | original_shape_Sphericity_ADC |
| ADC | First order | original_firstorder_Energy_ADC |
| ADC | First order | original_firstorder_InterquartileRange_ADC |
| ADC | First order | original_firstorder_TotalEnergy_ADC |
| ADC | Second order | original_glcm_ClusterProminence_ADC |
| ADC | Second order | original_glcm_Imc1_ADC |
| ADC | Second order | original_gldm_SmallDependenceEmphasis_ADC |
| ADC | Second order | original_glszm_HighGrayLevelZoneEmphasis_ADC |
| ADC | Second order | original_glszm_SizeZoneNonUniformityNormalized_ADC |
| ADC | Second order | original_glszm_SmallAreaHighGrayLevelEmphasis_ADC |
| ADC | Second order | original_glszm_ZonePercentage_ADC |
| ADC | Second order | original_ngtdm_Busyness_ADC |
| ADC | Second order | original_ngtdm_Strength_ADC |
| DWI | Second order | original_gldm_DependenceNonUniformityNormalized_DWI |
| DWI | Second order | original_gldm_GrayLevelVariance_DWI |
| DWI | Second order | original_gldm_LargeDependenceLowGrayLevelEmphasis_DWI |
| DWI | Second order | original_ngtdm_Coarseness_DWI |
| FLAIR | First order | original_firstorder_10Percentile_FLAIR |
| FLAIR | First order | original_firstorder_Kurtosis_FLAIR |
| FLAIR | First order | original_firstorder_Median_FLAIR |
| FLAIR | Second order | original_glszm_SmallAreaEmphasis_FLAIR |
| FLAIR | Second order | original_glszm_SmallAreaHighGrayLevelEmphasis_FLAIR |
| FLAIR | Second order | original_ngtdm_Busyness_FLAIR |
| FLAIR | Second order | original_ngtdm_Contrast_FLAIR |
| FLAIR | Second order | original_ngtdm_Strength_FLAIR |

ADC, apparent diffusion coefficient; DWI, diffusion weighted imaging; FLAIR, fluid attenuated inversion recovery; glcm, gray-level cooccurrence matrix; gldm, gray-level difference matrix; glrlm, gray-level run-length matrix; glszm, gray-level size zone matrix; ngtdm, neighbourhood gray-tone difference matrix.

# Supplementary Table 11. The Spearman correlation between radiomics features with mask ADC620 and 3-month mRS score

| **Feature Name** | **Correlation Coefficient** | ***p* value** |
| --- | --- | --- |
| original_shape_Elongation_ADC | 0.189 | 0.003 |
| original_shape_Maximum2DDiameterColumn_ADC | 0.494 | <0.001 |
| original_shape_Maximum2DDiameterRow_ADC | 0.401 | <0.001 |
| original_firstorder_Energy_ADC | 0.422 | <0.001 |
| original_firstorder_InterquartileRange_ADC | 0.152 | 0.017 |
| original_glcm_ClusterProminence_ADC | 0.267 | <0.001 |
| original_glcm_Imc1_ADC | -0.255 | <0.001 |
| original_gldm_SmallDependenceHighGrayLevelEmphasis_ADC | -0.191 | 0.003 |
| original_glrlm_ShortRunEmphasis_ADC | -0.231 | <0.001 |
| original_glszm_SizeZoneNonUniformity_ADC | 0.499 | <0.001 |
| original_glszm_SizeZoneNonUniformityNormalized_ADC | -0.041 | 0.521 |
| original_glszm_SmallAreaEmphasis_ADC | 0.328 | <0.001 |
| original_ngtdm_Strength_ADC | -0.364 | <0.001 |
| original_glrlm_GrayLevelNonUniformityNormalized_DWI | -0.478 | <0.001 |
| original_glrlm_GrayLevelVariance_DWI | 0.478 | <0.001 |
| original_glszm_GrayLevelNonUniformityNormalized_DWI | -0.443 | <0.001 |
| original_glszm_SizeZoneNonUniformity_DWI | 0.404 | <0.001 |
| original_glszm_SmallAreaLowGrayLevelEmphasis_DWI | 0.250 | <0.001 |
| original_ngtdm_Complexity_DWI | 0.473 | <0.001 |
| original_ngtdm_Contrast_DWI | 0.476 | <0.001 |
| original_ngtdm_Strength_DWI | 0.419 | <0.001 |
| original_firstorder_10Percentile_FLAIR | 0.007 | 0.916 |
| original_firstorder_Entropy_FLAIR | 0.339 | <0.001 |
| original_firstorder_Median_FLAIR | 0.014 | 0.827 |
| original_glcm_Contrast_FLAIR | 0.363 | <0.001 |
| original_gldm_LargeDependenceLowGrayLevelEmphasis_FLAIR | -0.116 | 0.068 |
| original_glszm_HighGrayLevelZoneEmphasis_FLAIR | 0.339 | <0.001 |
| original_ngtdm_Strength_FLAIR | 0.369 | <0.001 |

ADC, apparent diffusion coefficient; DWI, diffusion weighted imaging; FLAIR, fluid attenuated inversion recovery; glcm, gray-level cooccurrence matrix; gldm, gray-level difference matrix; glrlm, gray-level run-length matrix; glszm, gray-level size zone matrix; mRS, modified Rankin scale; ngtdm, neighbourhood gray-tone difference matrix.


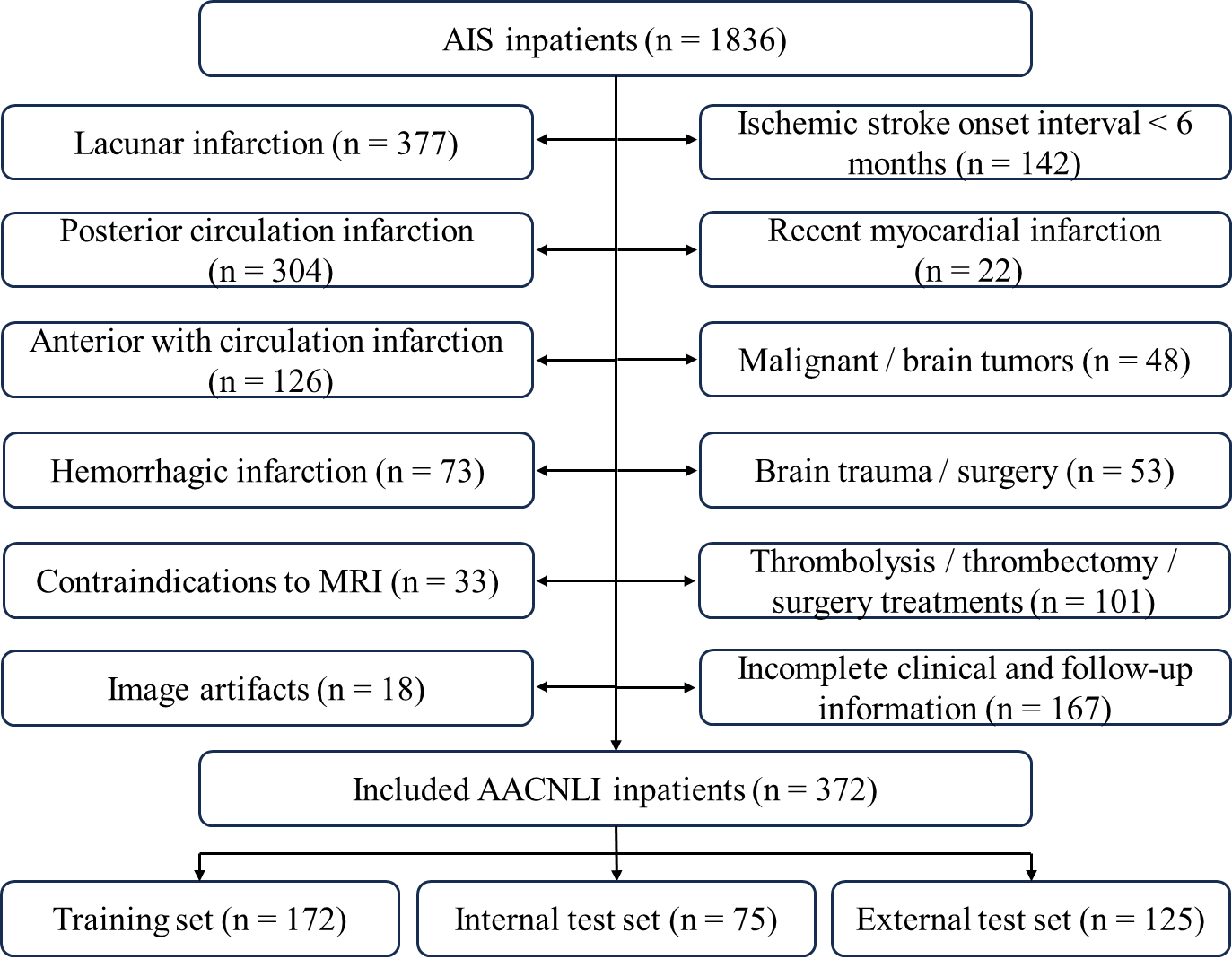


# Supplementary Figure 1. Flow chart illustrating AACNLI patients’ selection. Note: AACNLI, acute anterior circulation non-lacunar infarction; AIS, acute ischemic stroke.


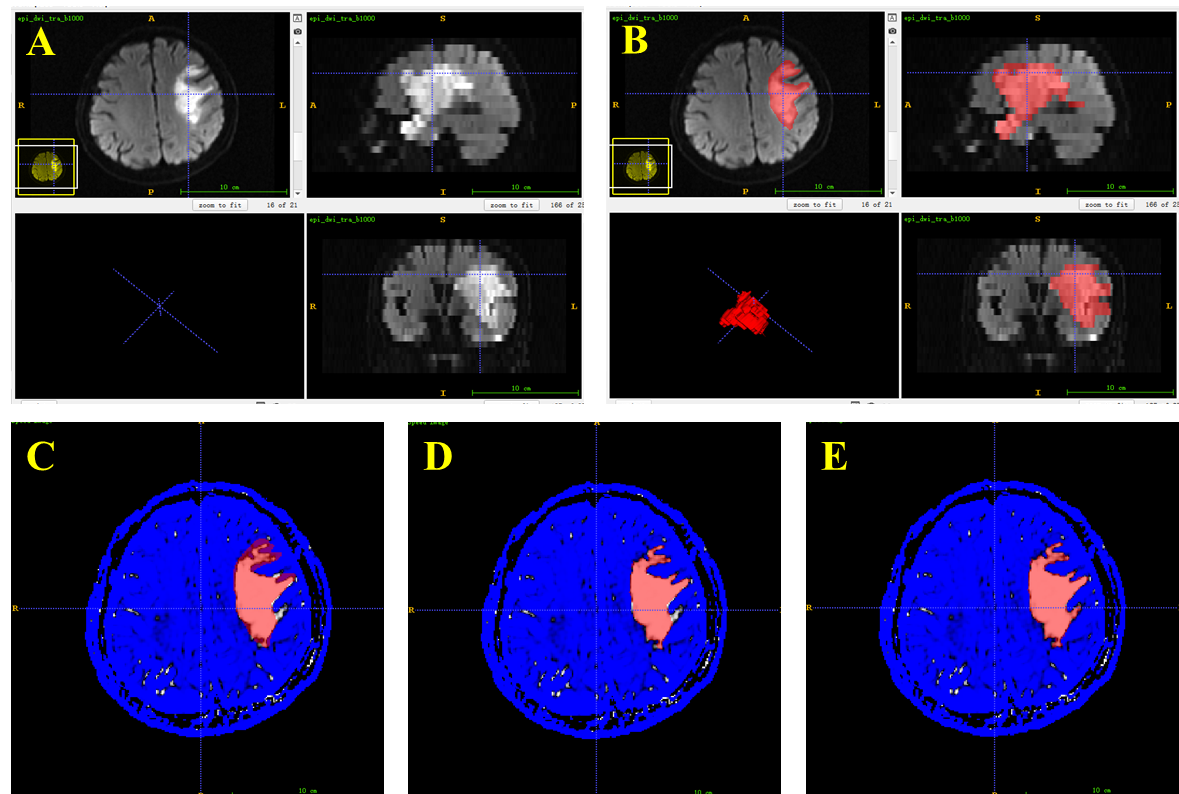


**Supplementary Figure 2. Segmentation results of mask DWI, mask ADC and mask ADC620.** The high-signal lesion of ischemic stroke on (A) DWI is segmented by ITK-SANP software (version 3.8.0), and (B) mask DWI is derived (red region). (C) The mask DWI is matched on ADC images, and ADC threshold of 0-620 is set (white region), while the voxels with ADC > 620 is shown as blue region. (D) The mask ADC is derived from the overlapped region with mask DWI and area of ADC 0-620. (E) The mask ADC620 is derived from the mask ADC and its adjacent area of ADC 0-620. Note: ADC, apparent diffusion coefficient; DWI, diffusion weighted imaging.


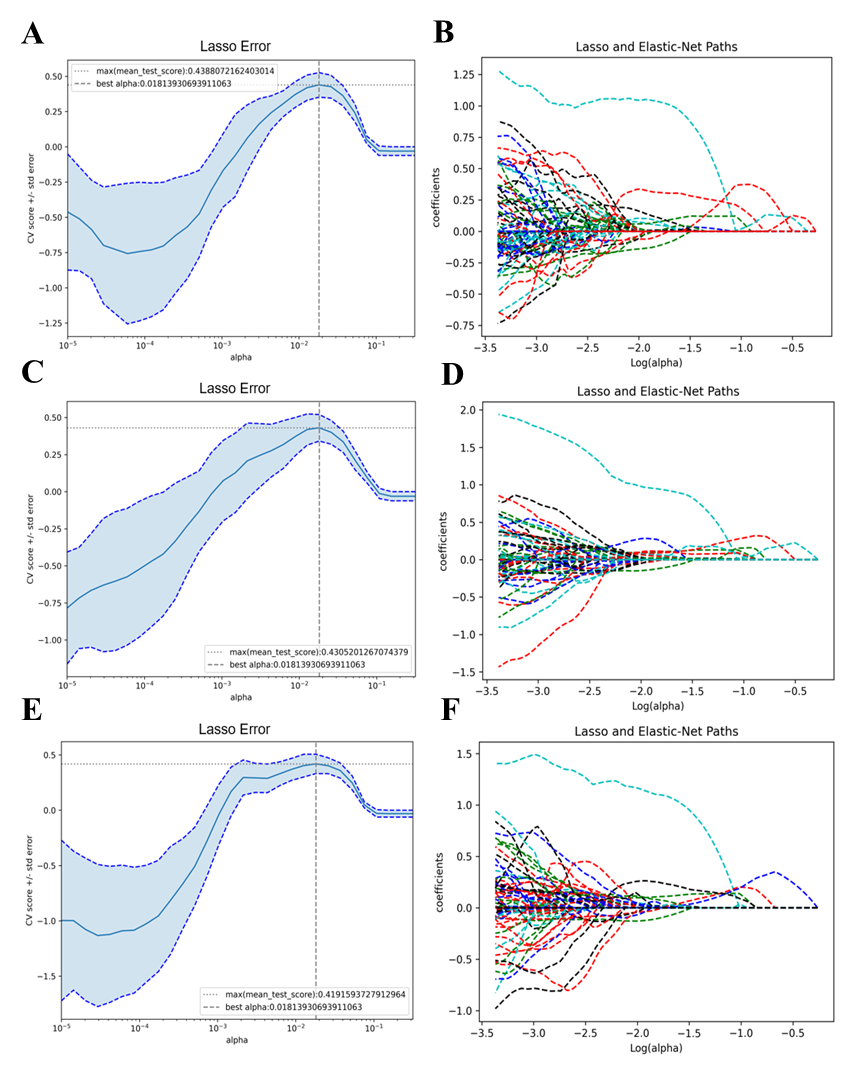


**Supplementary Figure 3. LASSO errors and LASSO paths of three types of masks.** (**A-B**) mask DWI, (**C-D**) mask ADC620, and (**E-F**) mask ADC. Note: ADC, apparent diffusion coefficient; DWI, diffusion weighted imaging; LASSO, least absolute shrinkage and selection operator.
